# Integrated Mendelian Randomization and Single-Cell Transcriptomics Reveal T Cell Immune Mechanisms in Systemic Lupus Erythematosus–Bladder Cancer Comorbidity

**DOI:** 10.3390/life16081381

**Published:** 2026-08-21

**Authors:** Desheng Zhang, Huan Ren, Yunjin Bai, Ping Han

**Affiliations:** 1 Department of Urology, West China Hospital, Sichuan University, Chengdu 610041, China; 2 Institute of Urology, West China Hospital, Sichuan University, Chengdu 610041, China

**Keywords:** systemic lupus erythematosus, bladder cancer, comorbidity mechanism, Mendelian randomization, single-cell transcriptome sequencing

## Abstract

**Objective:** Patients with systemic lupus erythematosus (SLE) exhibit elevated malignancy risk, with increased bladder cancer incidence. This study integrated Mendelian randomization (MR) with single-cell sequencing (scRNA-seq) to nominate exploratory prioritized candidate genes in SLE–bladder cancer comorbidity and their T cell regulatory roles. **Methods:** Single-cell datasets for SLE (GSE266852) and bladder cancer (GSE222315) were retrieved from GEO. Quality control, clustering, and annotation were performed using Seurat. T cell differentially expressed genes were intersected for bidirectional two-sample MR using IEU Open GWAS statistics. Heterogeneity, pleiotropy, and sensitivity analyses assessed robustness. GeneMANIA, miRNA databases, and CTD were used for network and functional analyses. Wilcoxon tests and Monocle 2 were used to characterize expression and T cell differentiation trajectories. **Results:** Cross-disease intersection nominated 1010 candidate genes. In an exploratory MR screen (uncorrected *p* < 0.05), four candidate genes were nominated (GBP3, LMAN1, SLC40A1, MIS18BP1); none survived FDR correction in both directions. At the uncorrected threshold, LMAN1 showed a shared risk direction (OR > 1) and MIS18BP1 a protective direction (OR < 1). Both showed significant T cell differential expression (*p* < 0.001) and elevated late differentiation expression. LMAN1 was involved in COPII vesicle transport; MIS18BP1 in CENP-A chromatin assembly. Twenty high-confidence miRNAs targeted each gene. CTD indicated liver injury associations and cisplatin/cyclosporine interactions. **Conclusions:** LMAN1 and MIS18BP1 are proposed as hypothesis-generating exploratory candidate genes in SLE–bladder cancer comorbidity, potentially involved in immune dysregulation through T cell terminal differentiation modulation. This study provides preliminary evidence suggestive of autoimmune–malignancy comorbidity mechanisms.

## 1. Introduction

Systemic lupus erythematosus (SLE) is a chronic autoimmune disease characterized by loss of immune tolerance and multi-system inflammatory damage, with a global prevalence of approximately 20–150 per 100,000 population. It predominantly affects women of childbearing age, with a female-to-male ratio of approximately 9:1 [1,2]. The pathogenesis of SLE remains incompletely elucidated; current evidence suggests that genetic susceptibility (e.g., HLA-DR2 and HLA-DR3 alleles, and non-HLA loci such as IRF5 and STAT4), epigenetic modifications, environmental triggers (ultraviolet exposure, viral infections, and certain drugs), and sex hormone regulation interactively contribute to disease initiation and progression [3]. At the immunological level, the core pathological features of SLE include excessive activation of the type I interferon signaling pathway, aberrant B cell proliferation with massive autoantibody production, T cell subset imbalance (such as regulatory T cell dysfunction and effector T cell hyperactivation), and immune complex-mediated complement system activation, ultimately leading to multi-organ tissue damage [4,5]. Although glucocorticoids combined with immunosuppressants remain the mainstay of current therapeutic strategies, some patients exhibit refractory disease courses or develop irreversible organ damage, with standardized mortality rates still 2–3 times higher than those of the general population [6,7], underscoring the clinical importance of deeply dissecting SLE immune dysregulation mechanisms and identifying novel intervention targets.

Bladder cancer is the tenth most common malignancy worldwide, with approximately 614,000 new cases and 220,000 deaths reported globally in 2022. The incidence in men is 3–4 times higher than in women, with smoking and occupational aromatic amine exposure being the principal risk factors [8]. At the molecular level, bladder carcinogenesis involves the accumulation of multi-gene mutations and aberrant epigenetic regulation, wherein driver events such as TERT promoter mutations, TP53 loss, and FGFR3 activating mutations lead to cell cycle dysregulation and apoptosis resistance [9]. Regarding the tumor microenvironment, bladder cancer is characterized by substantial immune cell infiltration; however, effector T cells frequently exhibit a functionally exhausted state with high PD-1 expression, while elevated proportions of regulatory T cells (Tregs) and tumor-associated macrophages collectively establish an immunosuppressive microenvironment that promotes tumor immune evasion [10,11]. Therefore, in-depth characterization of T cell immune microenvironment features in bladder cancer and identification of novel regulatory targets hold significant clinical implications for improving immunotherapy efficacy.

Multiple epidemiological studies have confirmed that SLE patients exhibit a significantly elevated risk of malignancy compared with the general population, with an overall cancer risk increase of approximately 62%. Notably, the SIR for bladder cancer reaches 1.66 (95% CI 1.07–2.59) [12], and the bladder cancer risk among Chinese SLE patients far exceeds that observed in other regions globally [13]. Beyond long-term immunosuppressive therapy (particularly cyclophosphamide exposure) and chronic inflammation-driven DNA damage accumulation, SLE and bladder cancer share deep immunoregulatory commonalities—both involve T cell subset imbalance, aberrant activation of the type I interferon signaling pathway, and impaired immune surveillance function, suggesting that the two diseases may share certain molecular pathological mechanisms. This provides a hypothetical framework for exploring candidate genes from the perspective of the T cell immune microenvironment.

Although epidemiological and immunological evidence suggests shared molecular mechanisms between SLE and bladder cancer, direct investigations into the exploratory prioritized candidate genes for this comorbidity remain scarce. Wang et al. (2024) identified shared differentially expressed genes and immune infiltration features via bulk transcriptomics [13], but bulk data cannot resolve cell-type-specific heterogeneity or determine the direction of association. Beyond this specific disease pair, integrative genetic analyses have increasingly explored comorbidity mechanisms between autoimmunity and malignancy. Wei et al. (2023) conducted a bidirectional Mendelian randomization study of gastric cancer and twelve autoimmune diseases [14], Zhan et al. (2024) identified TET2 as a shared gene signature linking atopic dermatitis to colorectal cancer through multi-omics integration [15], and Li et al. (2024) combined Mendelian randomization with immune cell profiling to reveal cervical cancer-associated genes in distinct immune populations [16]. Bernal-Bello et al. (2025) reviewed common immunopathological pathways linking autoimmunity to malignancy [17], yet their framework lacks genetic association inference and single-cell empirical support. While the integrative framework of MR and scRNA-seq has emerged in recent cancer and immunological studies, its application to dissect the SLE–bladder cancer comorbidity from both genetic association and single-cell resolution perspectives remains to be reported. Mendelian randomization uses genetic variants as instrumental variables to assess genetically predicted associations with disease risk, while single-cell RNA sequencing overcomes bulk limitations by reconstructing differentiation trajectories and capturing dynamic gene expression changes at single-cell resolution. Therefore, we combine these two approaches to provide cross-dimensional insights into the autoimmunity–malignancy comorbidity.

This study integrates Mendelian randomization with scRNA-seq to identify SLE–bladder cancer candidate genes. Bidirectional two-sample MR screened candidate genes using T cell differentially expressed gene intersections as exposures, with single-cell characterization performed using public datasets (GSE266852, GSE222315). Functional enrichment, network construction, miRNA prediction, and pseudotime analysis characterized their roles in T cell differentiation. This work provides genetic and cytological insights into comorbidity mechanisms and cross-disease intervention strategies.

## 2. Materials and Methods

### 2.1. Data Source

The study workflow is illustrated in Supplementary Materials S2. Single-cell datasets for SLE (GSE266852; 14,344 cells, 17,400 genes) and bladder cancer (GSE222315; 96,017 cells, 29,540 genes) were retrieved from GEO. Bidirectional two-sample MR was performed using the intersection of T cell DEGs as exposure variables, with SLE (finn-b-L12_LUPUS) and bladder cancer (ieu-b-4874) GWAS data from IEU OpenGWAS as outcomes.

### 2.2. Single-Cell Data Processing

Raw expression matrices were converted into Seurat objects (v5.4.0; min.cells = 3, min.features = 200) [18]. QC retained cells with 200–5000 detected genes and percent.mt < 15%. Data were normalized (LogNormalize, scale.factor = 10,000), variable genes selected (vst, top 2000), and standardized through ScaleData [19]. Principal component analysis (PCA) extracted 20 PCs, with optimal PCs determined by ElbowPlot. FindNeighbors, FindClusters, and uniform manifold approximation and projection (UMAP) projection completed dimensionality reduction and clustering [20]. Cell identities were annotated using canonical markers and CellMarker2.0 [21], yielding 10 populations: T cells, B cells, NK cells, monocytes, dendritic cells, endothelial cells, fibroblasts, macrophages, smooth muscle cells, and plasma cells. Compositional differences between cases and controls identified target cell types for downstream analysis.

### 2.3. Identification of DEGs and Enrichment Analysis

DEG screening for each cell subtype was performed using the FindMarkers function in the Seurat package. The SLE dataset threshold was set at |log_2_FC| > 0.25 and *p* < 0.05, while the bladder cancer dataset concurrently adopted |log_2_FC| > 0.25 and *p* < 0.05. Cross-disease shared molecular features were identified by intersecting T cell DEGs from both diseases, and the resulting candidate gene set was used for downstream in-depth analysis. Functional annotation was conducted using the clusterProfiler package (v4.6.0) [22], encompassing three Gene Ontology (GO) branches (BP, CC, MF) and Kyoto Encyclopedia of Genes and Genomes (KEGG) signaling pathway enrichment, with *p* < 0.05 as the significance threshold for each term.

### 2.4. Mendelian Randomization Analysis for Candidate Gene Identification

The validity of Mendelian randomization is contingent upon three core assumptions: (1) the instrumental variables (IVs) are strongly associated with the exposure (relevance assumption); (2) the IVs are independent of confounders that affect the exposure–outcome association (exchangeability assumption); and (3) the IVs affect the outcome only through the exposure, without horizontal pleiotropic effects independent of the exposure (exclusion restriction assumption). For the exposure variable, cis-eQTL summary statistics were obtained from the eQTLGen Consortium (https://www.eqtlgen.org/) (accessed on 16 August 2025) through the IEU OpenGWAS database (accession IDs: eqtl-a-ENSG00000000003 to eqtl-a-ENSG00000270149). SNP instruments were selected at genome-wide significance (*p* < 5 × 10^−8^) and clumped for linkage disequilibrium (R^2^ < 0.001 within a 10,000 kb window, following IEU OpenGWAS standard preprocessing parameters) to ensure independence among retained SNPs. The F-statistic was calculated for each SNP as F = β^2^/SE^2^ (where β and SE represent the effect size and standard error of the SNP–gene expression association, respectively), and only SNPs with F > 10 were retained as strong instruments. After this rigorous screening, valid instrumental variables for 14,312 genes were obtained. The intersection of these eQTL-derived genes with T-cell differentially expressed genes (DEGs) from both SLE and bladder cancer scRNA-seq datasets served as the final exposure variable set for MR analysis. This two-step filtering strategy—intersecting T-cell DEGs from both diseases prior to MR—was used as a screening and prioritization tool, not as a confirmatory analysis. By restricting MR to cross-disease DEGs in T cells, we aimed to reduce noise and alleviate the multiple-testing burden of genome-wide MR. However, this pre-filtering may exclude population-relevant causal genes that do not meet T-cell DEG thresholds. Thus, the nominated genes should be viewed as prioritized candidates requiring independent validation, and future genome-wide MR without such pre-filtering would be valuable.

It should be noted that eQTL-based MR uses genetically regulated transcriptional levels as the exposure variable; however, mRNA abundance does not linearly correspond to protein abundance or functional activity (with a typical mRNA–protein Pearson correlation of r ≈ 0.4–0.6 in mammalian cells) [23]. Therefore, MR estimates should be interpreted as effects of “genetically predicted gene expression” rather than definitive protein-level genetically predicted associations. Two-sample MR analysis was implemented using the TwoSampleMR package (v0.5.6) in R, with GWAS summary statistics for both exposure and outcome derived from the IEU OpenGWAS public repository. The intersection of SLE–bladder cancer T cell DEGs served as the exposure variable, with SLE and bladder cancer as independent outcome variables, respectively, to bidirectionally assess genetically predicted associations. To enhance reliability, five methods were simultaneously employed: Inverse Variance Weighted (IVW) [24], MR-Egger [25], weighted median [26], simple mode [27] and weighted mode [28], with IVW as the primary effect estimate and *p* < 0.05 considered indicative of a genetically predicted association. Effect sizes were presented as odds ratios (OR), with OR > 1 suggesting a risk effect and OR < 1 a protective effect. Genes reaching nominal significance (*p* < 0.05, uncorrected) in both directions of MR were incorporated into the comorbidity exploratory-prioritized-candidate-gene pool. For statistical diagnostics, mr_heterogeneity assessed instrumental variable heterogeneity, mr_pleiotropy_test screened for horizontal pleiotropy bias, mr_leaveoneout performed leave-one-out robustness testing, and MR-Steiger evaluated directional consistency.

### 2.5. Network Construction, miRNA Prediction, and Drug Interaction Analysis

The functional interaction landscape of candidate genes was constructed through the GeneMANIA platform (https://genemania.org) (accessed on 27 March 2026), with emphasis on identifying neighboring genes exhibiting physical interactions or co-expression relationships with LMAN1 and MIS18BP1, and parsing their GO functional clustering characteristics. At the post-transcriptional regulatory level, prediction results from miRWALK (http://mirwalk.umm.uni-heidelberg.de) (accessed on 6 May 2026) and miRDB (http://www.mirdb.org) (accessed on 6 May 2026) were integrated, and high-confidence targeting miRNAs were obtained by intersection; representative molecules were extracted and ranked by regulatory weight for network construction. For disease association, the CTD database (http://ctdbase.org) (accessed on 6 May 2026) was utilized to quantify the association strength between candidate genes and phenotypes, and disease lineages with top inference scores were selected for visualization. Furthermore, based on the CTD chemical exposure module, the interaction profiles of LMAN1 and MIS18BP1 with environmental toxicants and clinical drugs were systematically catalogued, providing leads for subsequent mechanistic interpretation and intervention target screening.

### 2.6. Cell Type Localization and Pseudotime Analysis Methods

Cellular localization of candidate genes was determined by Wilcoxon rank-sum test, comparing expression differences between cases and controls within each cell subtype in SLE (GSE266852) and bladder cancer (GSE222315), respectively. Subtypes reaching significance in both directions (*p* < 0.05) were designated as target cell types. For this cell population, the Monocle 2 package was employed to reconstruct differentiation trajectories: secondary clustering via UMAP was first performed to subdivide subpopulations, and FindAllMarkers was used to extract cluster-specific markers; subsequently, DDRTree dimensionality reduction constructed the pseudotime axis, and orderCells was applied to infer state transitions [29]. The BEAM model further resolved branch-point genes, and plot_pseudotime_heatmap was used to present temporal expression heatmaps of candidate genes, thereby suggesting potential dynamic regulatory patterns of LMAN1 and MIS18BP1 during disease progression.

### 2.7. Statistical Analysis

All computational workflows were executed in R v4.5.0, with core analyses relying on Seurat v5.4.0, Monocle 2 v2.36.0, clusterProfiler v4.6.0, TwoSampleMR v0.5.6, and ggplot2 v4.0.2. All computational workflows were executed in R 4.5.0, with core analyses relying on Seurat 5.4.0 and its extended package suite. Two-sided tests were used for all group comparisons, with *p* < 0.05 as the significance threshold. Visualization outputs were generated using the R base graphics system combined with ggplot2, and final manuscript layouts were refined in Adobe Illustrator 2023. To control the false-positive risk arising from multiple hypothesis testing, we performed false discovery rate (FDR) correction on the *p*-values obtained from the inverse variance weighted (IVW) method in the Mendelian randomization analysis. The Benjamini–Hochberg method was applied separately to the two independent MR directions (systemic lupus erythematosus and bladder cancer). The significance threshold after FDR correction was set at q < 0.05. Sensitivity analyses (including heterogeneity testing, pleiotropy testing, leave-one-out analysis, and Steiger directionality testing) were used to evaluate the robustness of individual MR estimates but were not considered substitutes for multiple-testing correction. The complete FDR-corrected *p*-values for all candidate genes in both directions are provided in Supplementary Table S8.

## 3. Results

### 3.1. Single-Cell Transcriptomic Characterization of SLE

**Figure 1:**
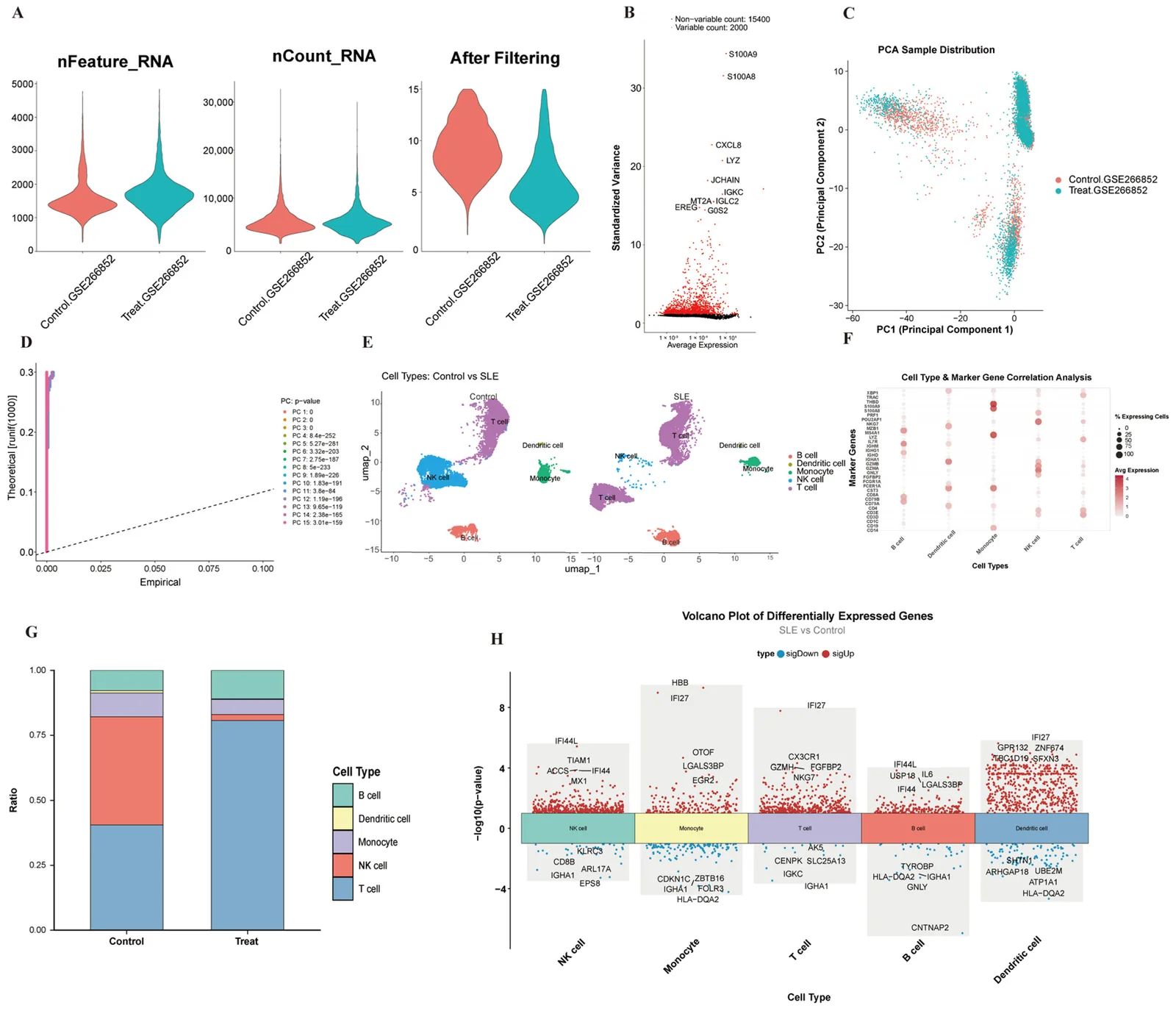
Single-cell RNA sequencing analysis shows cellular heterogeneity and differential expression characteristics in SLE. (**A**) Quality control metrics showing nFeature_RNA, nCount_RNA, and gene counts after filtering for control and SLE groups. (**B**) Gene expression feature scatter plot showing variable gene selection (vst, top 2000), with the top 10 most variable genes labeled. (**C**) PCA sample distribution between control and SLE groups. (**D**) JackStraw analysis demonstrating the statistical significance of principal components; the first 12 PCs reached significance (*p* < 0.05). (**E**) UMAP clustering showing 12 cell clusters (left) and distribution between control and SLE groups (right). (**F**) Cell type–specific marker gene correlation analysis. (**G**) Comparison of cell type proportions between control and SLE groups. (**H**) Volcano plots of differentially expressed genes across the five annotated cell types in SLE.

Following QC, GSE266852 retained 14,344 cells (7506 control, 6838 SLE) and 17,400 genes (Figure 1A). Variable feature selection (vst, top 2000) and top 10 annotation were performed (Figure 1B). PCA showed no obvious group separation (Figure 1C). JackStraw analysis showed that the first 12 principal components reached statistical significance (*p* < 0.05), explaining the major variation in the data; therefore, these 12 PCs were selected for subsequent dimensionality reduction and clustering (Figure 1D). Clustering identified 12 clusters (Figure 1E), annotated as five cell types: B cells, dendritic cells, monocytes, NK cells, and T cells (Figure 1E,F). SLE showed increased monocytes, decreased T/NK cells, and emergent dendritic cells (Figure 1G), indicating innate immune activation and adaptive suppression. DEG analysis identified 3960 SLE genes (union across types): B cells (257 up, 129 down), dendritic cells (1 up), monocytes (445 up, 195 down), NK cells (504 up, 104 down), and T cells (2949 up, 470 down) (Figure 1H). These findings suggest that SLE is characterized by a profound imbalance between innate and adaptive immunity, with T cells exhibiting the most extensive transcriptional reprogramming. Given that T cell dysfunction is a shared pathological axis in both SLE and malignancy, the large T cell DEG repertoire (2949 upregulated genes) provides a robust candidate pool for cross-disease intersection and subsequent genetically predicted association screening.

### 3.2. Single-Cell Data Analysis of Bladder Cancer

**Figure 2:**
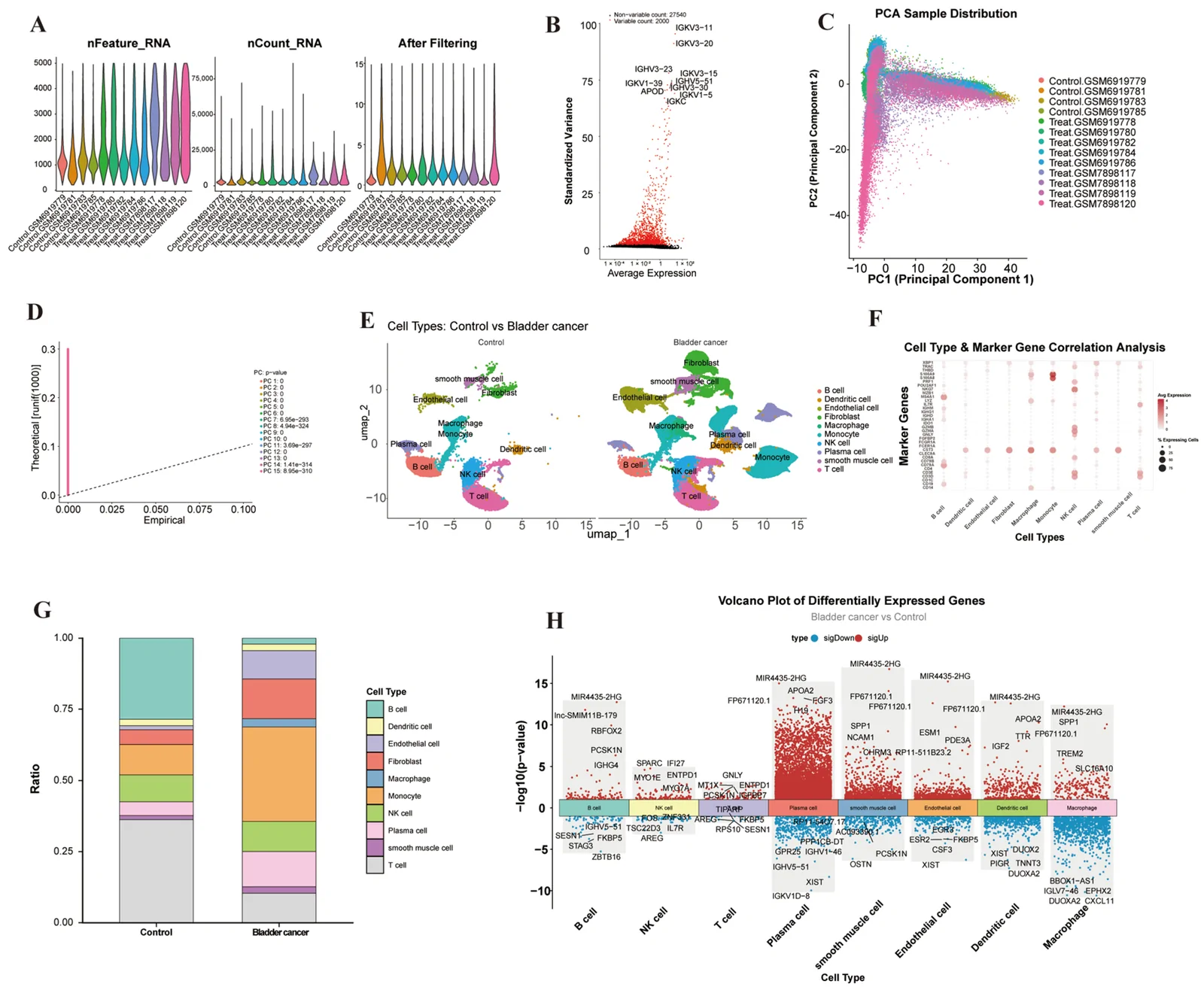
Single-cell RNA sequencing analysis shows cellular heterogeneity and differential expression characteristics in bladder cancer. (**A**) Quality control metrics showing nFeature_RNA, nCount_RNA, and gene counts after filtering for control and bladder cancer groups. (**B**) Gene expression feature scatter plot showing variable gene selection (vst, top 2000), with the top 10 most variable genes labeled. (**C**) PCA sample distribution between control and bladder cancer groups. (**D**) JackStraw analysis demonstrating the statistical significance of principal components; the first 15 PCs reached significance (*p* < 0.05). (**E**) UMAP clustering showing 25 cell clusters (left) and distribution between control and bladder cancer groups (right). (**F**) Cell type–specific marker gene correlation analysis. (**G**) Comparison of cell type proportions between control and bladder cancer groups. (**H**) Volcano plots of differentially expressed genes across the 10 annotated cell types in bladder cancer.

After QC, GSE222315 retained 96,017 cells (24,933 control, 71,084 bladder cancer) and 29,540 genes (Figure 2A). Variable feature selection (vst, top 2000) and top 10 annotation were performed (Figure 2B). PCA showed no obvious group separation (Figure 2C). JackStraw analysis showed that the first 15 principal components reached statistical significance (*p* < 0.05), explaining the major variation in the data; therefore, these 15 PCs were selected for subsequent dimensionality reduction and clustering (Figure 2D). Clustering identified 25 cell clusters (Figure 2E), annotated as 10 cell types: B cells, dendritic cells, endothelial cells, fibroblasts, macrophages, monocytes, NK cells, plasma cells, smooth muscle cells, and T cells (Figure 2E,F). Bladder cancer showed decreased B cells and increased fibroblasts, monocytes, and plasma cells compared with controls (Figure 2G), indicating tumor microenvironment remodeling with enhanced stromal and innate immune infiltration. DEG analysis identified 16,363 BC genes (union across 10 cell types): B cells (945 up, 453 down), dendritic cells (846 up, 2344 down), endothelial cells (573 up, 581 down), fibroblasts (3960 up, 2136 down), macrophages (2 up, 557 down), monocytes (8776 up, 2745 down), NK cells (1048 up, 749 down), plasma cells (9450 up, 1376 down), smooth muscle cells (2457 up, 259 down), and T cells (1806 up, 766 down) (Figure 2H). Notably, bladder cancer also exhibits substantial T cell transcriptional alterations (1806 upregulated genes), suggesting that T cell dysregulation is not unique to autoimmunity but represents a convergent pathological feature across both diseases. This shared T cell-centric molecular signature motivated the subsequent cross-disease intersection to identify comorbidity candidate genes operating within the T cell compartment.

### 3.3. Cross-Disease T Cell DEG Intersection and Functional Enrichment

**Figure 3:**
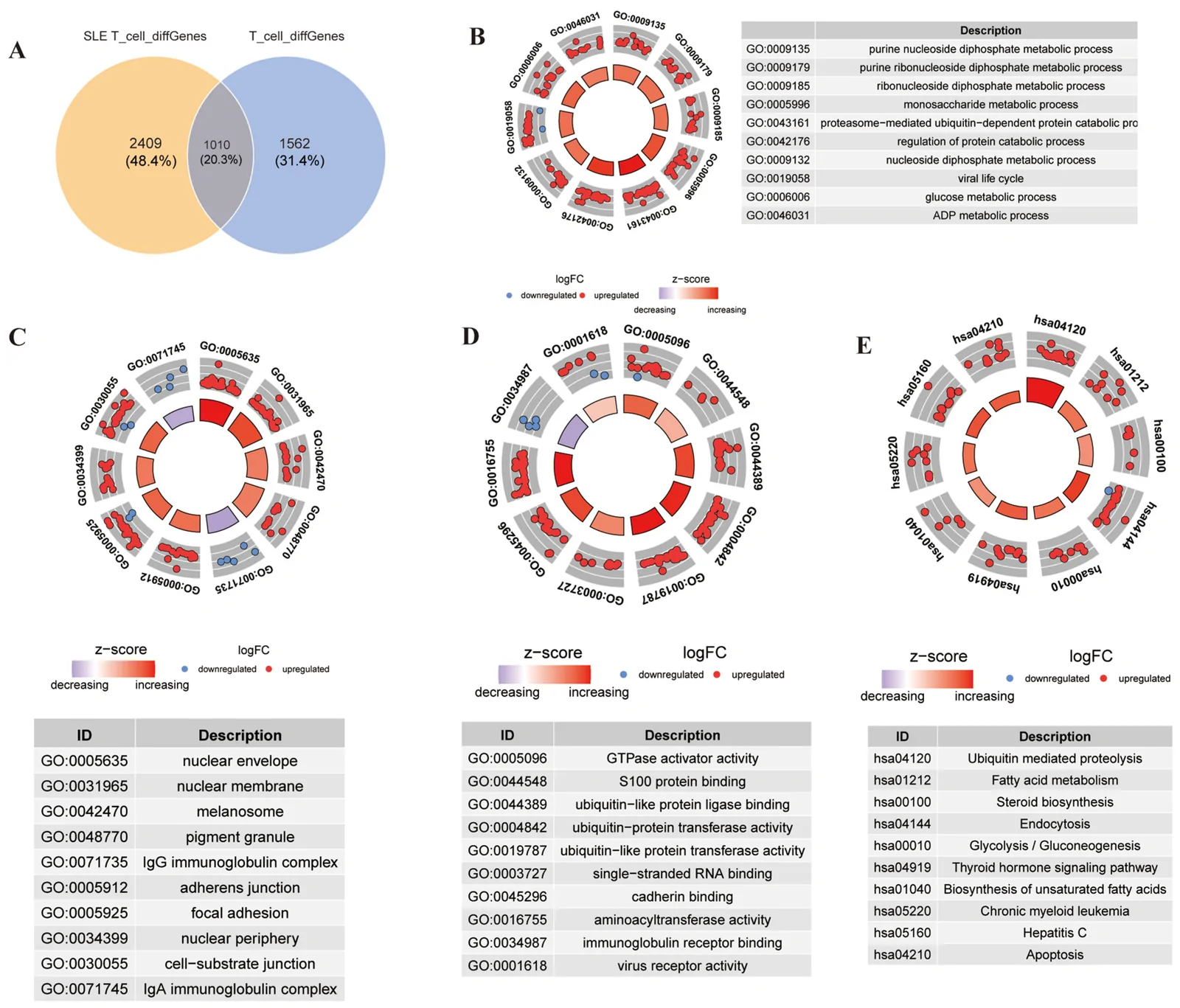
Cross-disease T cell differentially expressed gene intersection and functional enrichment. (**A**) Venn diagram showing the intersection of T cell DEGs between SLE and bladder cancer, yielding 1010 shared candidate genes. (**B**) Gene Ontology (GO) biological process (BP) enrichment circle plot of the 1010 intersecting genes. (**C**) GO cellular component (CC) enrichment circle plot. (**D**) GO molecular function (MF) enrichment circle plot. (**E**) Kyoto Encyclopedia of Genes and Genomes (KEGG) pathway enrichment circle plot. Color intensity reflects enrichment significance and fold change direction (red = upregulated, blue = downregulated).

Cross-disease T cell DEG intersection nominated 1010 candidate genes shared between SLE and bladder cancer (Figure 3A). Functional enrichment of these intersecting genes revealed 1033 significant GO terms: 830 biological process (BP), 107 molecular function (MF), and 96 cellular component (CC) terms (Figure 3B–D). Representative BP terms included purine nucleoside diphosphate metabolic process, ribonucleoside diphosphate metabolic process, monosaccharide metabolic process, proteasome-mediated ubiquitin-dependent protein catabolic process, and glucose metabolic process (Figure 3B). Key CC terms encompassed nuclear envelope, nuclear membrane, melanosome, pigment granule, IgG immunoglobulin complex, adherens junction, focal adhesion, and nuclear periphery (Figure 3C). Significant MF terms included GTPase activator activity, S100 protein binding, ubiquitin-protein transferase activity, ubiquitin-like protein ligase binding, single-stranded RNA binding, and immunoglobulin receptor binding (Figure 3D). KEGG pathway analysis identified 41 significant pathways, including ubiquitin mediated proteolysis, fatty acid metabolism, steroid biosynthesis, endocytosis, glycolysis/gluconeogenesis, and apoptosis (Figure 3E). Collectively, these enrichment patterns suggest that the shared T cell transcriptomic signature in SLE and bladder cancer converges on metabolic reprogramming (purine, fatty acid, and glucose metabolism), protein homeostasis disturbance (ubiquitin-proteasome system), and altered membrane trafficking and signal transduction (endocytosis, GTPase regulation, and cell–cell adhesion). These findings provide a biologically grounded candidate pool for subsequent Mendelian randomization, implicating that T cell metabolic and post-translational dysregulation may constitute a shared pathological axis underlying autoimmune–malignancy comorbidity.

### 3.4. MR Analysis for Candidate Gene Identification

**Figure 4:**
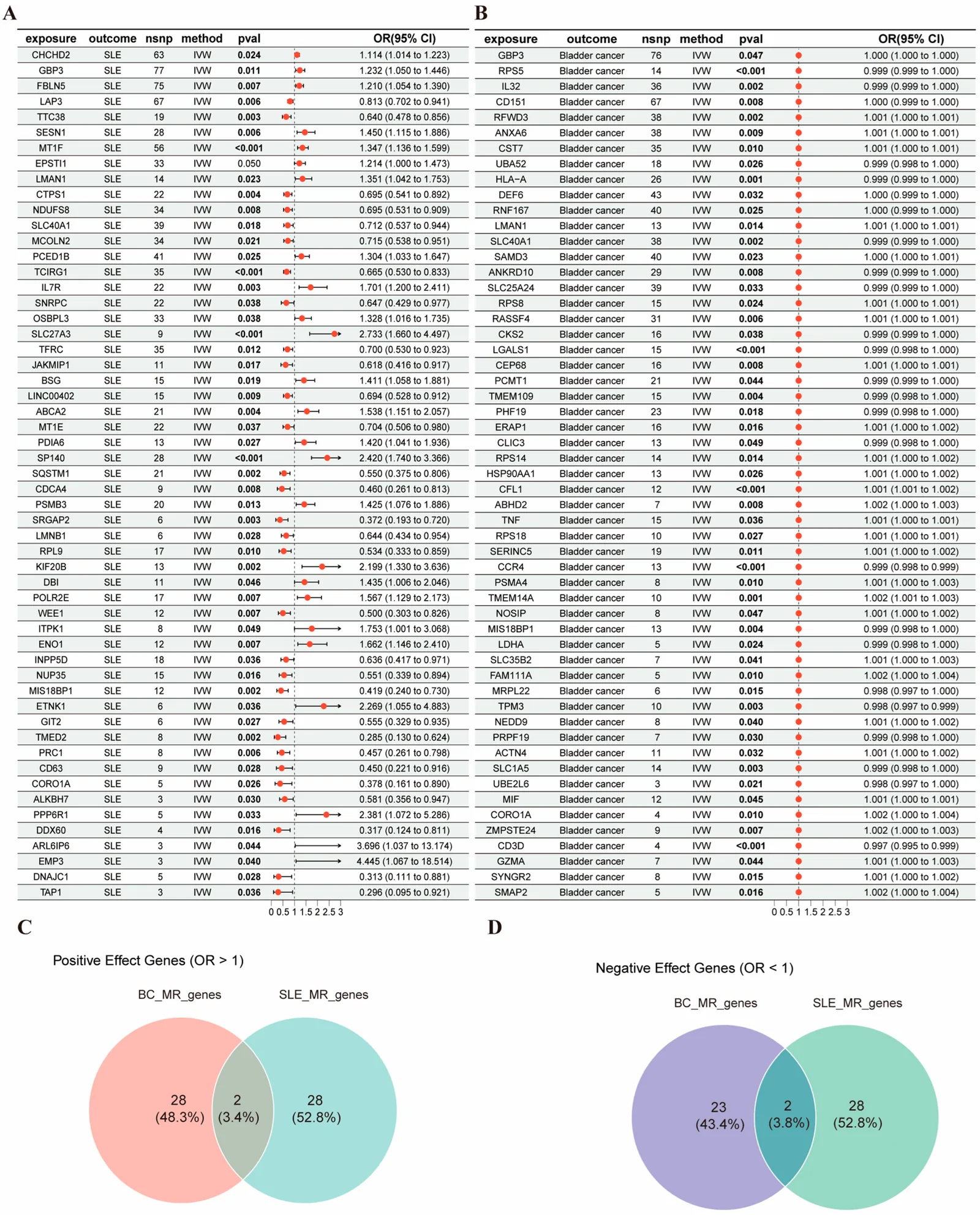
Mendelian randomization screening of cross-disease candidate genes. (**A**) Forest plot showing the complete bidirectional MR results (IVW method) for 55 genes associated with systemic lupus erythematosus (SLE), with effect sizes expressed as odds ratios (OR) and 95% confidence intervals. Genes are ranked by effect direction and magnitude; the top 10 risk-direction genes (OR > 1) and bottom 10 protective-direction genes (OR < 1) are highlighted. (**B**) Forest plot showing the complete MR results for 55 genes associated with bladder cancer. (**C**) Venn diagram of positive-effect genes (OR > 1) shared between SLE and bladder cancer. (**D**) Venn diagram of negative-effect genes (OR < 1) shared between SLE and bladder cancer. The intersection in each Venn diagram represents genes with concordant effect directions across both diseases.

At the uncorrected *p* < 0.05 threshold, MR analysis nominated 55 genes with evidence of genetically predicted association with SLE and 55 with bladder cancer (Figure 4A,B). The intersection yielded four candidate genes: GBP3, LMAN1, SLC40A1, and MIS18BP1 (Figure 4C,D). However, after FDR correction (Benjamini–Hochberg, q < 0.05), no gene remained significant in both directions; LMAN1 and MIS18BP1 did not survive multiplicity correction in either direction. Despite this, we retained LMAN1 and MIS18BP1 for downstream single-cell characterization based on two considerations: (i) their biological relevance to T cell terminal differentiation (COPII vesicle transport and CENP-A chromatin assembly, respectively), which aligns with the functional enrichment patterns of the cross-disease T cell DEGs (Figure 3B–E); and (ii) their significant differential expression in T cells in both diseases (Figures 8A and 9A), providing orthogonal non-genetic evidence. These genes are therefore interpreted as exploratory, hypothesis-generating prioritized candidates rather than statistically robust causal genes. LMAN1 and MIS18BP1 were prioritized from the four candidate genes for in-depth downstream analysis based on their putative biological relevance to T cell terminal differentiation. LMAN1-mediated COPII vesicle transport governs glycosylation-dependent trafficking of immune receptors (including TCR and co-stimulatory molecules) to the cell surface, thereby regulating immunological synapse formation and T cell activation thresholds [30,31]. MIS18BP1-mediated CENP-A chromatin assembly has been implicated in epigenetic remodeling programs that may influence effector versus memory T cell fate decisions, as chromatin accessibility at relevant loci (IFNG, TBX21, FOXP3) is a core determinant of T cell differentiation [32,33]. By contrast, GBP3 is primarily implicated in innate immunity and interferon-driven inflammatory responses [34], whereas SLC40A1 (ferroportin) governs systemic iron homeostasis and ferroptosis susceptibility [35]; although iron metabolism indirectly influences immune effector function, SLC40A1’s established role is rooted in metabolic regulation rather than the direct epigenetic or membrane-trafficking mechanisms [36] that govern T cell differentiation trajectories. Consequently, LMAN1 and MIS18BP1—representing mechanistically distinct yet putatively relevant pathways—were selected to explore how vesicle transport and chromatin dynamics converge on T cell dysfunction in both diseases. Notably, the functional enrichment of the 1010 cross-disease T cell DEGs provided independent data-driven support for this prioritization: GO analysis revealed significant enrichment of nuclear envelope and nuclear membrane terms (Figure 3C), consistent with MIS18BP1’s chromatin assembly function, whereas KEGG nominated endocytosis (Figure 3E) and GO highlighted GTPase activator activity (Figure 3D), aligning with LMAN1’s vesicle transport role. Furthermore, the convergent enrichment of ubiquitin-proteasome system terms across GO BP, MF, and KEGG (Figure 3B–E) reflects shared protein quality control mechanisms underlying both genes.

**Figure 5:**
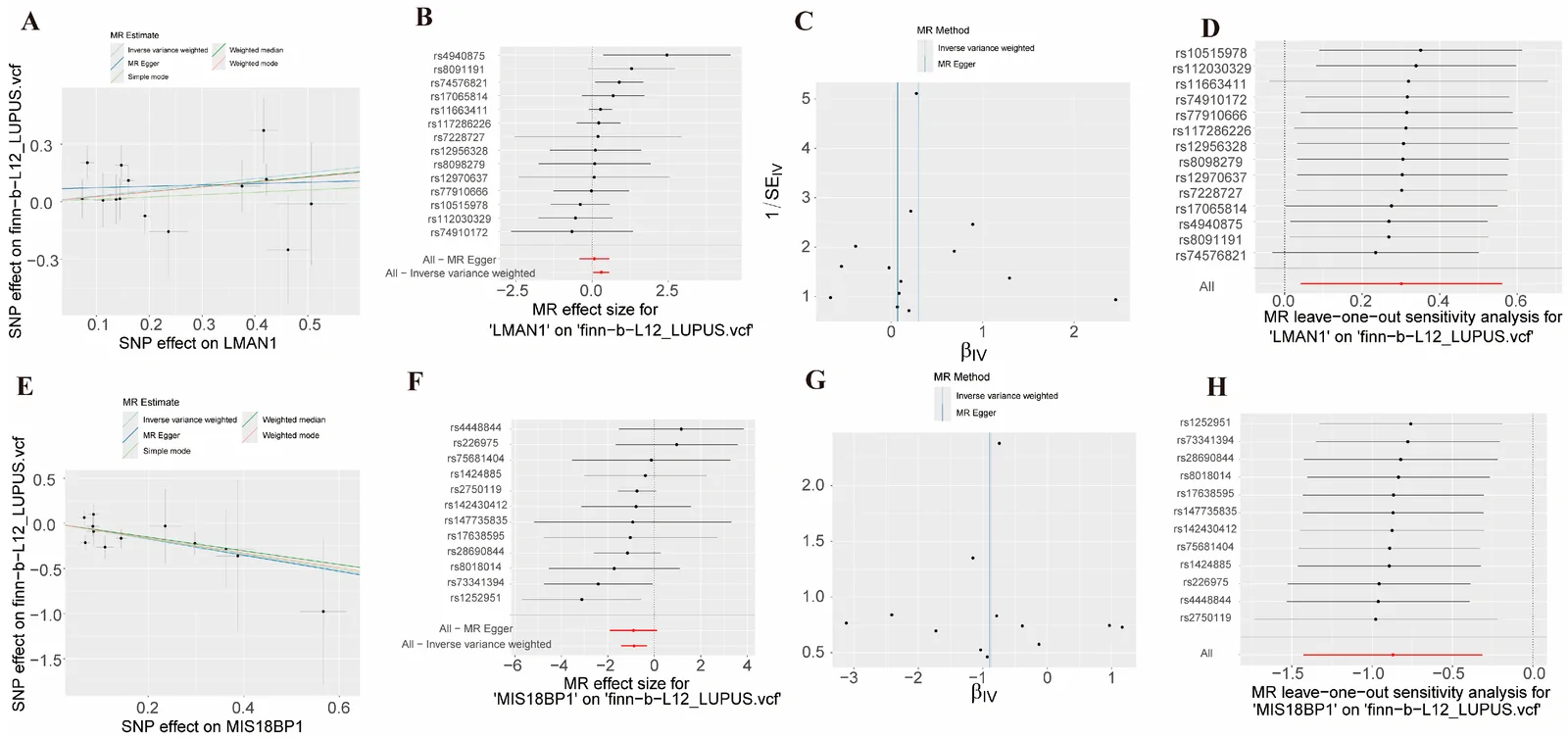
Sensitivity analyses of LMAN1 and MIS18BP1 with systemic lupus erythematosus (SLE) in Mendelian randomization. (**A**–**D**) LMAN1: scatter plot of SNP effects on LMAN1 versus SNP effects on SLE (**A**), forest plot of individual SNP and combined MR estimates (**B**), funnel plot of 1/SE against MR effect size (**C**), and leave-one-out sensitivity analysis (**D**). (**E**–**H**) MIS18BP1: scatter plot (**E**), forest plot (**F**), funnel plot (**G**), and leave-one-out analysis (**H**). All analyses were performed using the TwoSampleMR package with inverse variance weighted (IVW), MR-Egger, weighted median, simple mode, and weighted mode methods.

**Figure 6:**
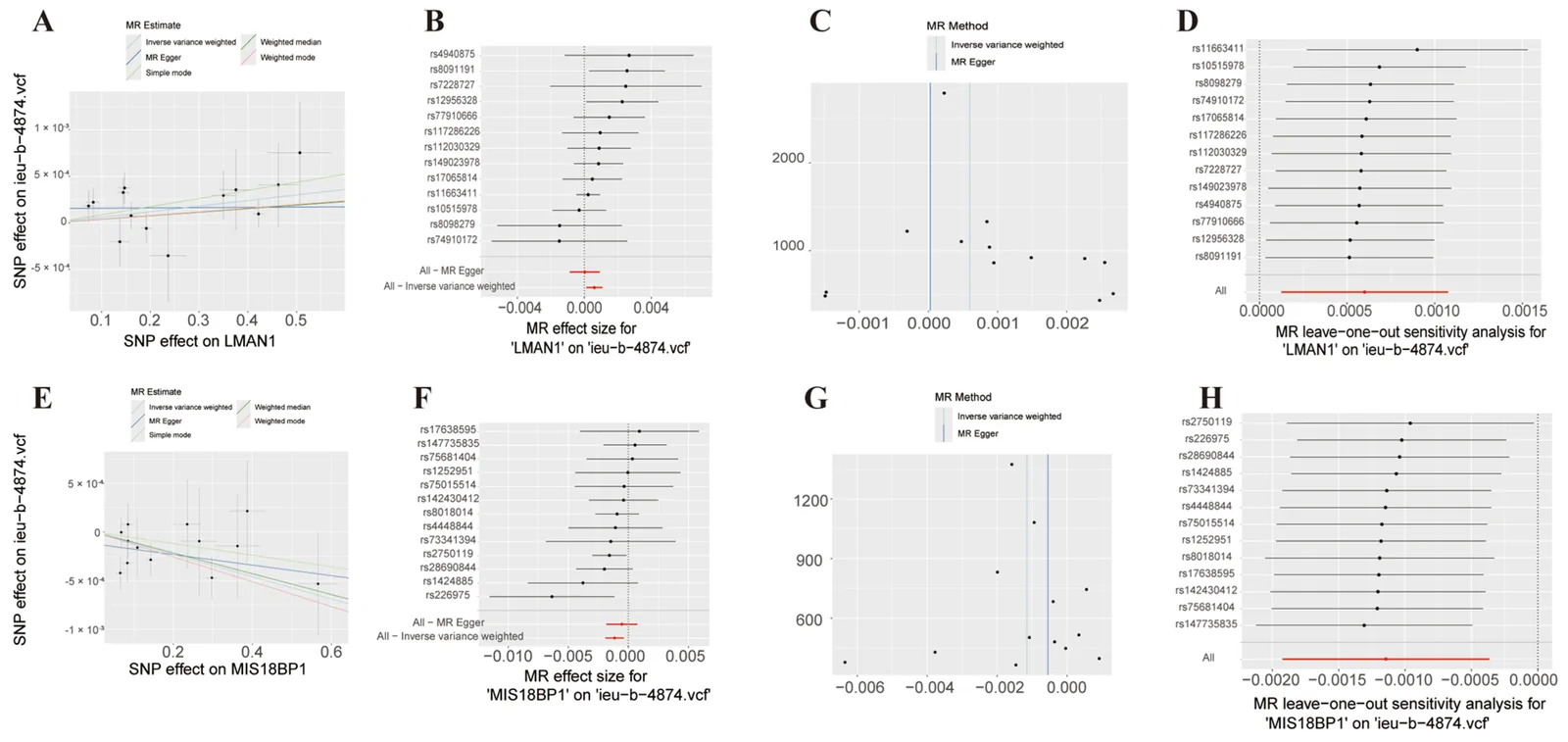
Sensitivity analyses of LMAN1 and MIS18BP1 with bladder cancer in Mendelian randomization. (**A**–**D**) LMAN1: scatter plot of SNP effects on LMAN1 versus SNP effects on bladder cancer (**A**), forest plot (**B**), funnel plot (**C**), and leave-one-out analysis (**D**). (**E**–**H**) MIS18BP1: scatter plot (**E**), forest plot (**F**), funnel plot (**G**), and leave-one-out analysis (**H**).

Bidirectional MR with SLE as outcome (55 genes) showed no significant heterogeneity (Table S1), no significant pleiotropy (Table S2), and consistent directionalities (Steiger TRUE, Table S3). Supplemental Tables S1–S3 provide detailed statistics that further assess the reliability of the instrumental variables. Cochran’s Q test indicated no significant heterogeneity across all 55 genes (all *p* > 0.05). MR-Egger intercept tests revealed no evidence of directional pleiotropy (e.g., LMAN1: *p* = 0.298; MIS18BP1: *p* = 0.959). Furthermore, Steiger directionality tests were consistent with a direction from gene expression to SLE for all genes (all *p* < 1 × 10^−86^). LMAN1-SLE and MIS18BP1-SLE analyses showed positive scatter trends, consistent forest plots, symmetric funnels, and robust leave-one-out results (Figure 5A–H). Bidirectional MR with bladder cancer as outcome (55 genes) showed no heterogeneity (Table S4), no pleiotropy (Table S5), and consistent directions (Steiger TRUE, Table S6). Supplemental Tables S4–S6 also support the robustness of the MR results in the bladder cancer direction. Cochran’s Q statistics showed no significant heterogeneity in all tests, MR-Egger intercept tests yielded *p*-values > 0.05 for all pleiotropy assessments, and Steiger directionality tests were consistent with the hypothesized directionality (all *p* < 1 × 10^−139^) MIS18BP1-bladder cancer and LMAN1-bladder cancer analyses showed consistent scatter, forest, funnel, and leave-one-out results (Figure 6A–H).

### 3.5. Regulatory Network Analysis and Drug Prediction for Candidate Genes

**Figure 7:**
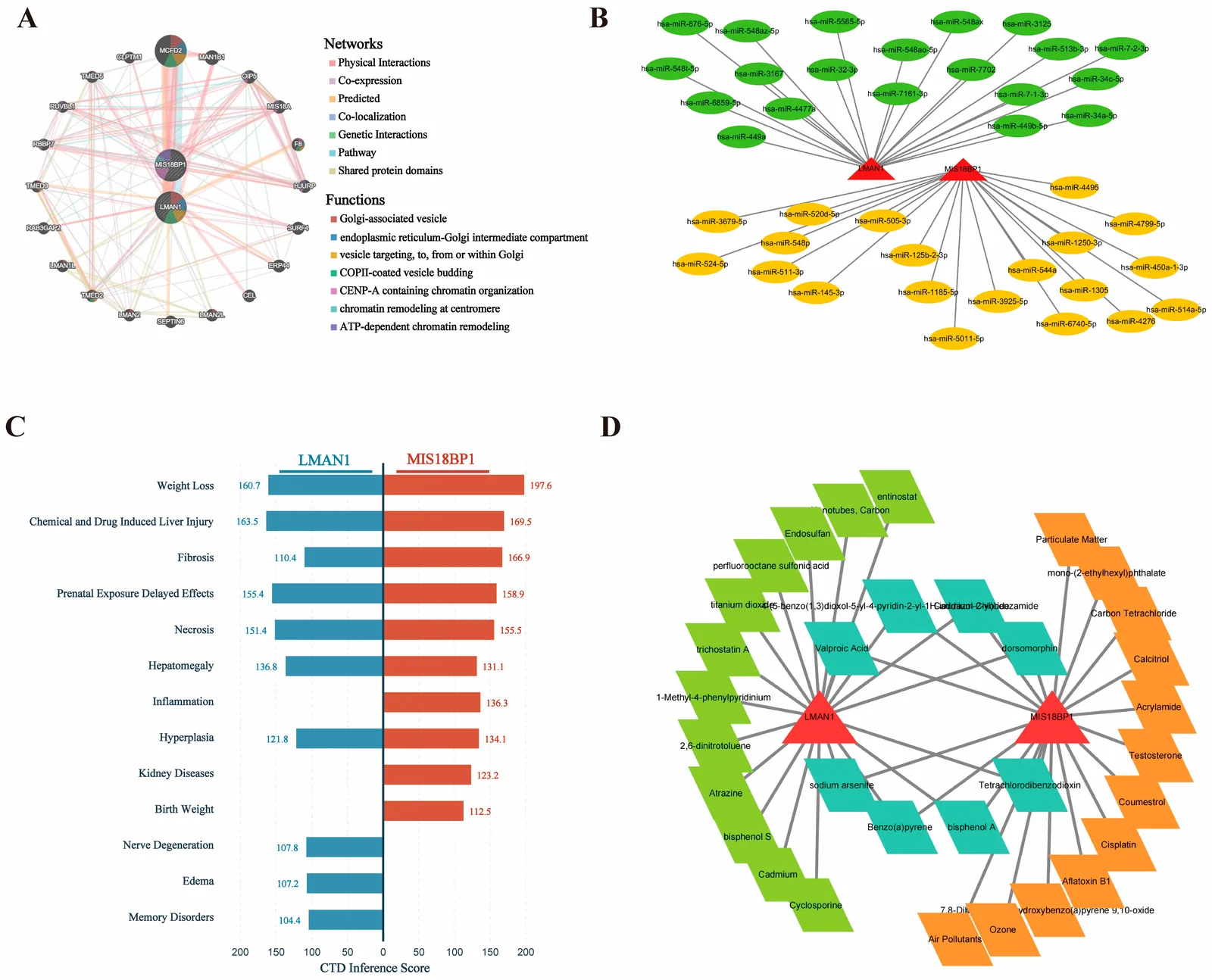
Regulatory network analysis and drug prediction for candidate genes. (**A**) GeneMANIA protein–protein interaction network showing functional associations, interaction types (physical interactions, co-expression, predicted, co-localization, genetic interactions, pathway, shared protein domains), and functional annotations of LMAN1 and MIS18BP1 with neighboring genes. (**B**) miRNA regulatory network showing high-confidence miRNAs targeting LMAN1 (green nodes) and MIS18BP1 (yellow nodes), with candidate genes (LMAN1 and MIS18BP1) shown as red triangles. (**C**) CTD-based gene–disease association analysis comparing the inference scores of LMAN1 (leftward blue bars) and MIS18BP1 (rightward red bars) across specific diseases (butterfly plot). (**D**) Chemical–gene interaction network showing environmental toxicants and drugs potentially interacting with LMAN1 and/or MIS18BP1. Compounds are color-coded by shared status: green = LMAN1 only, orange = MIS18BP1 only, cyan = both genes.

GeneMANIA networks identified LMAN1-interactors (MCFD2, MAN1B1, SURF4, ERP44, CEL, LMAN2) and MIS18BP1-interactors (OIP5, MIS18A, HJURP), enriched in Golgi vesicle transport, COPII budding, CENP-A chromatin assembly, and ATP-dependent chromatin remodeling (Figure 7A). miRNA network analysis identified 20 high-confidence miRNAs targeting each gene (top 5 by binding strength: LMAN1—hsa-miR-34a-5p, hsa-miR-449a, hsa-miR-32-3p, hsa-miR-548t-5p, hsa-miR-876-5p; MIS18BP1—hsa-miR-524-5p, hsa-miR-520d-5p, hsa-miR-505-3p, hsa-miR-145-3p, hsa-miR-511-3p) (Figure 7B). CTD analysis associated LMAN1 with chemically induced liver injury, fibrosis, necrosis, and hepatomegaly; MIS18BP1 with weight loss, fibrosis, inflammation, and kidney disease (Figure 7C). Both genes showed potential interactions with cisplatin, cyclosporine, aflatoxin B1, benzo(a)pyrene, and 32 additional environmental toxicants/drugs (Figure 7D). These network analyses suggest functionally divergent protein modules: LMAN1 is embedded in an endoplasmic reticulum–Golgi membrane-trafficking axis consistent with its genetically predicted risk effect, whereas MIS18BP1 is anchored to a centromeric chromatin-remodeling complex compatible with its protective role. The shared miRNA regulatory architecture and overlapping chemical interaction profiles (e.g., cisplatin and cyclosporine) suggest that post-transcriptional and environmental factors may converge to modulate T cell differentiation through these two distinct molecular axes, providing hypothetical entry points for cross-disease pharmacological intervention.

### 3.6. Identification of Potentially Relevant Cell Types and Pseudotime Analysis

**Figure 8:**
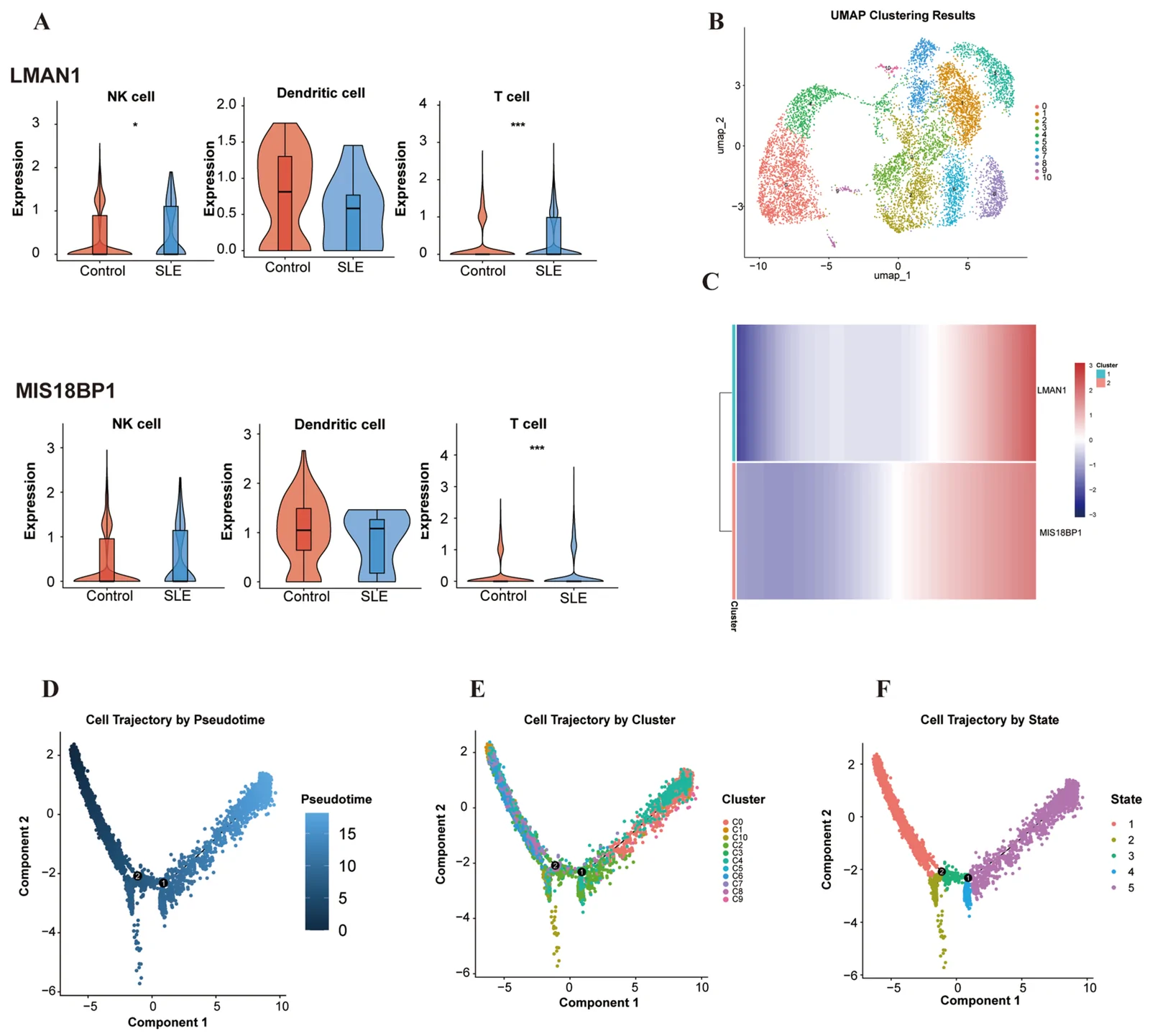
Expression characterization and pseudotime analysis of candidate genes in SLE. (**A**) Violin plots showing LMAN1 (upper) and MIS18BP1 (lower) expression across three immune cell types (NK cells, dendritic cells, and T cells) in control and SLE groups. T cells showed significant differential expression for both genes ((Wilcoxon test, *** adjusted *p* < 0.001; * adjusted *p* < 0.05 for LMAN1 in NK cells)). (**B**) UMAP projection of 11 T cell subpopulations (C0–C10). (**C**) Pseudotime expression heatmap of LMAN1 and MIS18BP1 (row-scaled Z-score). Both genes showed early-low, late-high expression patterns. (**D**–**F**) Monocle 2 DDRTree trajectory colored by pseudotime (**D**), cluster identity (**E**), and state (**F**).

To further characterize the expression characteristics of candidate genes across different cell types, we analyzed all samples from the GSE266852 dataset. Results showed that both LMAN1 and MIS18BP1 exhibited significantly differential expression in T cells (both *p* < 0.001), whereas differential expression was non-significant or only partially significant in NK cells and dendritic cells; therefore, T cells were designated as the potentially relevant cell population (Figure 8A). This cell-type-restricted expression pattern supports the biological plausibility of the MR findings by demonstrating that the genetically prioritized genes are transcriptionally active within the disease-relevant T cell compartment in SLE. Pseudotime analysis further showed the developmental trajectories of T cell subpopulations and the dynamic expression patterns of candidate genes. Based on the Monocle 2 algorithm, T cells from the GSE266852 dataset were ordered along pseudotime, identifying 11 T cell subpopulations (Figure 8B). The pseudotime trajectory showed a dynamic process of progressive T cell differentiation from early to late stages along the pseudotime axis. Both LMAN1 and MIS18BP1 exhibited early low expression and late high expression patterns along the pseudotime trajectory (Figure 8C), suggesting that these two genes may participate in late T cell activation or terminal differentiation stages. Further differential analysis of the 10 T cell subpopulations showed that subpopulation C6 (corresponding to the late pseudotime stage) exhibited significantly higher expression levels of LMAN1 and MIS18BP1 than other subpopulations (Figure 8D–F), identifying C6 as a subpopulation exhibiting markedly elevated candidate gene expression and suggesting that this subpopulation may play an important role in SLE-related T cell dysfunction. The progressive enrichment of both genes in late-pseudotime T cells suggests that their comorbidity-associated effects may be exerted during terminal effector differentiation, a stage characterized by persistent autoreactive activation in SLE.

**Figure 9:**
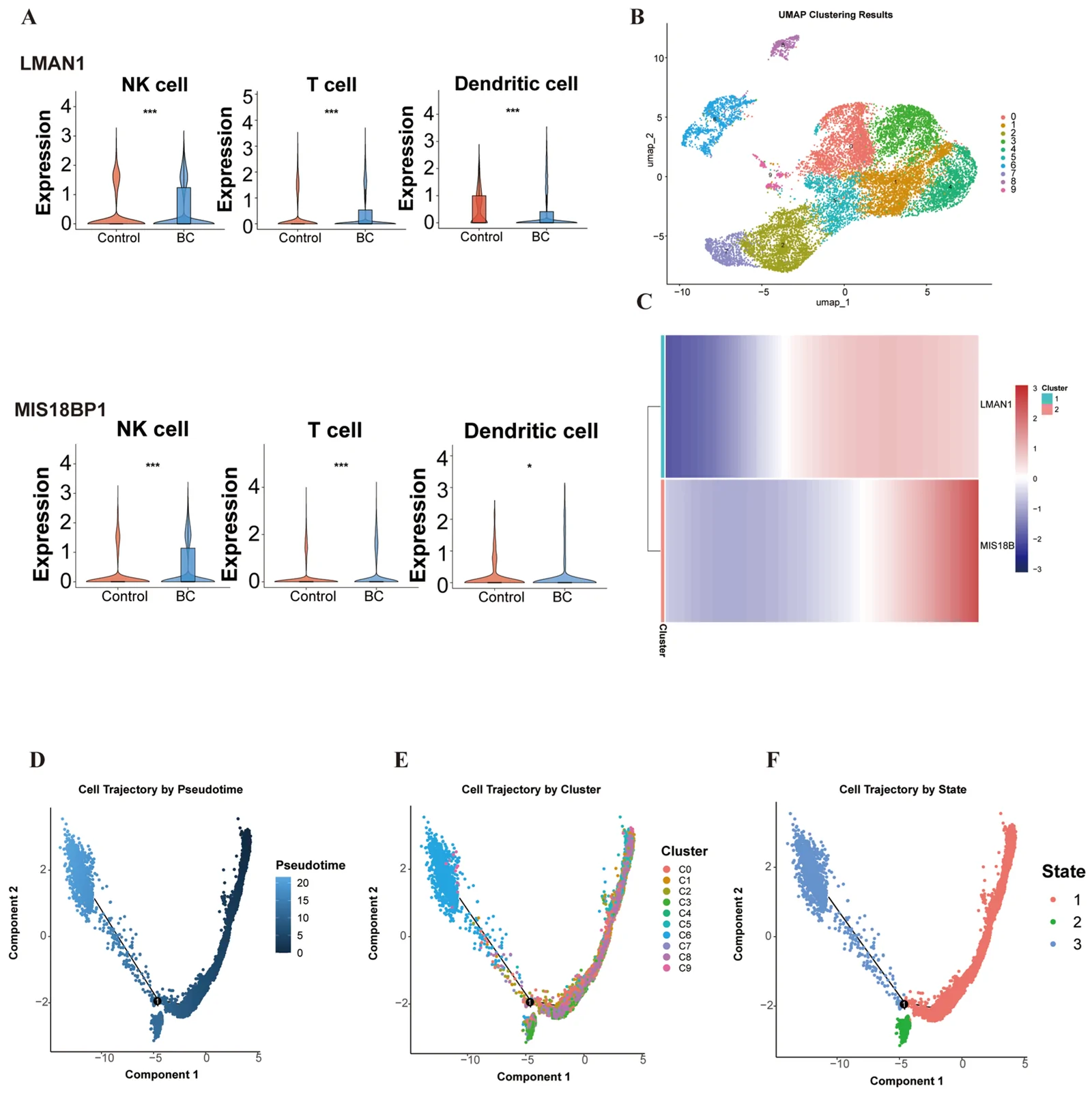
Expression characterization and pseudotime analysis of candidate genes in bladder cancer (GSE222315). (**A**) Violin plots showing LMAN1 (upper) and MIS18BP1 (lower) expression across three immune cell types (NK cells, dendritic cells, and T cells) in control and bladder cancer groups. T cells showed significant differential expression for both genes ((Wilcoxon test, *** adjusted *p* < 0.001; * adjusted *p* < 0.05)). (**B**) UMAP projection of 10 T cell subpopulations (C0–C9). (**C**) Pseudotime expression heatmap of LMAN1 and MIS18BP1 (row-scaled Z-score). Both genes showed early-low, late-high expression patterns. (**D**–**F**) Monocle 2 DDRTree trajectory colored by pseudotime (**D**), cluster identity (**E**), and state (**F**).

Wilcoxon analysis of GSE222315 showed that both LMAN1 and MIS18BP1 exhibited significantly differential expression in T cells (both *p* < 0.001), with markedly elevated expression in bladder cancer compared with controls (Figure 9A). The convergent T cell-specific differential expression across both diseases strengthens the hypothesis that these genes operate within a shared T cell-centric pathological axis. Pseudotime analysis further showed the developmental trajectories of T cell subpopulations and the dynamic expression patterns of candidate genes. Based on the Monocle 2 algorithm, T cells from the GSE222315 dataset were ordered along pseudotime, identifying 10 T cell subpopulations (Figure 9B). The pseudotime trajectory showed a dynamic process of progressive T cell differentiation from early to late stages along the pseudotime axis. Both LMAN1 and MIS18BP1 exhibited early low expression and late high expression patterns along the pseudotime trajectory (Figure 9C), suggesting that these two genes may participate in late T cell activation or terminal differentiation stages. Further differential analysis of the 9 T cell subpopulations showed that subpopulation C8 (corresponding to the late pseudotime stage) exhibited significantly higher expression levels of LMAN1 and MIS18BP1 than other subpopulations (Figure 9D–F), identifying C8 as a subpopulation exhibiting markedly elevated candidate gene expression and suggesting that this subpopulation may play an important role in bladder cancer-related T cell dysfunction. The parallel late-stage upregulation in bladder cancer T cells is consistent with the acquisition of an exhausted or terminally differentiated state within the tumor microenvironment, compatible with a shared temporal regulatory program across autoimmune and malignant T cell dysregulation.

## 4. Discussion

This study systematically explored the molecular mechanisms of SLE–bladder cancer comorbidity through the integration of Mendelian randomization and single-cell transcriptome sequencing analysis, from the dual perspectives of genetic association inference and cellular heterogeneity resolution. The study first nominated cross-disease T cell differentially expressed gene intersections, and subsequently nominated four exploratory candidate genes (GBP3, LMAN1, SLC40A1, and MIS18BP1) through bidirectional MR analysis at an uncorrected *p* < 0.05 threshold; none of these survived FDR correction in both directions. LMAN1 and MIS18BP1 were selected as representative candidate genes for in-depth analysis due to their genetically predicted associations in both diseases at the uncorrected *p* < 0.05 threshold, though these associations did not survive FDR correction for multiple testing. Single-cell data provided orthogonal evidence that LMAN1 and MIS18BP1 were significantly differentially expressed in T cell subpopulations in both SLE and bladder cancer, and pseudotime analysis revealed significantly elevated expression of both genes during late T cell differentiation stages, suggesting that they may participate in the immune dysregulation processes of both diseases through modulation of T cell terminal differentiation. This finding provides genetic and cytological leads for understanding the shared T cell immune pathological mechanisms in SLE and bladder cancer. Unlike prior bulk or review-based approaches [13] and genetic studies without cellular resolution [14,15,16], our MR–scRNA-seq integration nominates exploratory candidate genes and explores T cell dynamics, offering complementary insights into this comorbidity.

LMAN1 (ERGIC-53) is a type I transmembrane protein localized to the endoplasmic reticulum–Golgi intermediate compartment, with a luminal L-type lectin domain capable of binding mannose in a calcium-dependent manner. As a cargo receptor, LMAN1 forms a complex with MCFD2 to recruit and transport secretory proteins such as coagulation factors V and VIII from the endoplasmic reticulum to the Golgi apparatus via the COPII vesicle transport pathway; loss-of-function mutations can cause combined deficiency of coagulation factors V and VIII [37]. In this study, LMAN1 exhibited a genetically predicted risk direction (OR > 1) in both SLE and bladder cancer at the uncorrected threshold, though this association did not survive FDR correction, and GeneMANIA network analysis showed close interactions with endoplasmic reticulum–Golgi-related proteins such as MCFD2 and MAN1B1, with enriched functions concentrated in COPII vesicle budding and Golgi targeting transport. Based on these findings, we propose the following working hypothesis: LMAN1 may influence T cell activation thresholds and signal transduction intensity by regulating glycosylation modification and membrane trafficking efficiency of immune receptors (such as T cell receptors and co-stimulatory molecules). Previous studies have shown that LMAN1 not only participates in COPII vesicle transport of soluble secretory proteins, but also serves as a cargo receptor for membrane proteins, mediating their anterograde transport from the endoplasmic reticulum to the cell surface [30]. Furthermore, N-glycosylation of the T cell receptor has been demonstrated to dynamically regulate immunological synapse formation and signaling thresholds through galectin–glycoprotein lattices [31,38]. However, direct links between LMAN1 and immune receptor glycosylation remain to be experimentally validated. Therefore, this mechanism should be considered a reasonable hypothesis generated from our data, warranting functional validation in future studies. In SLE, excessive sensitivity of the T cell receptor signaling pathway is a central mechanism enabling autoreactive T cells to escape thymic selection; in bladder cancer, exhaustion of T cell effector function is closely associated with decreased efficiency of immunological synapse formation. Therefore, LMAN1-mediated vesicle transport abnormalities may represent a shared nodal point connecting T cell dysfunction in both diseases. Furthermore, CTD database predictions showed associations between LMAN1 and pathological processes including chemically induced liver injury and fibrosis, suggesting that it may play a broader role in tissue damage induced by chronic inflammatory microenvironments.

Pseudotime analysis further explored the pathological implications of the aforementioned expression dynamics. Quantitatively, pseudotime-binned expression analysis showed progressively increasing expression of both genes along the T cell differentiation trajectory, with expression levels in terminal differentiation subpopulations approximately 1.6-fold (LMAN1) and 2.7-fold (MIS18BP1) higher than in early-stage subpopulations in SLE, and 2.2-fold (LMAN1) and 2.5-fold (MIS18BP1) in bladder cancer (mean of pseudotime deciles 1–3 vs. 8–10; Supplementary Table S7), consistent with the heatmap visualization (Figure 8C and Figure 9C). In SLE, the late stage of the T-cell pseudotime trajectory (subpopulation C6) is inferred to correspond to the terminal differentiation state of effector T cells. A central immunopathological feature of SLE is aberrant T-cell receptor (TCR) signaling, characterized by downregulation of the CD3ζ chain and compensatory replacement with the FcRγ chain, which lowers the T-cell activation threshold and promotes persistent peripheral activation and differentiation of autoreactive T cells toward effector/memory phenotypes [39,40]. The markedly elevated expression of LMAN1 and MIS18BP1 in C6 suggests that these two genes may participate in the terminal differentiation program associated with SLE-related T-cell dysfunction. However, this pathological association currently remains a computational inference-based hypothesis, warranting validation in independent cohorts. In bladder cancer, the late stage of the pseudotime trajectory (subpopulation C8) is inferred to correspond to the exhausted state of T cells within the tumor microenvironment. The solid tumor microenvironment drives tumor-infiltrating T cells to progressively lose cytotoxic function, upregulate exhaustion markers such as PD-1 and TIM-3, and eventually enter terminal exhaustion through multiple signals including persistent antigen exposure, hypoxia, and accumulation of immunosuppressive metabolites [41,42]. This process involves profound epigenetic remodeling and metabolic reprogramming [43]. The elevated expression of LMAN1 and MIS18BP1 in C8 suggests their potential involvement in the epigenetic or metabolic reprogramming of this exhaustion program. Similarly, this interpretation remains a working hypothesis based on single-cell trajectory inference, warranting functional validation.

MIS18BP1 (MIS18 binding protein 1) is a core subunit of the Mis18 complex, which binds through its N-terminal domain to the Mis18α-Mis18β heterohexamer to form a heterooctamer, recruiting the CENP-A-specific chaperone HJURP at centromeres to complete CENP-A deposition. PLK1 activates this complex by recognizing the auto-phosphorylation sites Thr78 and Ser93 of MIS18BP1, thereby relieving the intramolecular inhibition of Mis18α and promoting HJURP binding to ensure faithful centromere inheritance during cell division [44]. In this study, MIS18BP1 exhibited a genetically predicted protective direction (OR < 1) in both SLE and bladder cancer at the uncorrected threshold, though this association did not survive FDR correction, a directional characteristic that contrasts sharply with LMAN1, suggesting that candidate genes may exhibit apparently opposing effect directions or environment-dependent bidirectional regulation. GeneMANIA analysis showed that MIS18BP1 interacts with chromosomal organization-related proteins such as HJURP and OIP5, with enriched functions concentrated in CENP-A chromatin assembly and ATP-dependent chromatin remodeling. In T cell biology, chromatin remodeling is a core determinant of T cell differentiation fate—the formation of effector T cells depends on chromatin opening at critical transcription factor loci (such as IFNG and TBX21), whereas Treg stability relies on epigenetic maintenance of the FOXP3 locus. Based on our current dataset, we hypothesize that MIS18BP1 may affect the balance of T cell subset differentiation through the regulation of chromatin structure. MIS18BP1, as a core component of the Mis18 complex, is known to be required for centromeric localization of CENP-A [45]. In addition, T cell fate decisions—including the stability of the regulatory T (Treg) cell lineage and the acquisition of effector T cell functions—are highly dependent on chromatin accessibility and epigenetic remodeling at critical gene loci [32,33]. Nevertheless, whether MIS18BP1 directly regulates chromatin accessibility at non-centromeric sites in T cells remains to be experimentally determined, and current data do not substantiate such a role. Thus, the proposed mechanism should be viewed as a reasonable hypothesis derived from our correlative data, and awaits rigorous functional validation. The feature of high MIS18BP1 expression in late-stage T cells observed in pseudotime analysis is consistent with its potential involvement in epigenetic reprogramming during terminal differentiation stages.

However, before further interpreting this protective direction, one apparent paradox merits explicit consideration. Canonically, MIS18BP1 functions as a core subunit of the Mis18 complex, orchestrating CENP-A deposition and centromere assembly during cell division—processes typically associated with proliferation. Intuitively, one might expect a gene linked to cell division to act as a risk factor in malignancy. However, our MR results indicate a protective direction of genetically predicted MIS18BP1 expression in both SLE and bladder cancer at the uncorrected threshold, though this association did not survive FDR correction. We propose two non-mutually exclusive interpretations. First, MIS18BP1 may exert its protective effect primarily through immune cells rather than through direct effects on tumor cell proliferation; in T cells, MIS18BP1-related chromatin remodeling may preserve epigenetic integrity during differentiation, thereby preventing aberrant effector programs in SLE or terminal exhaustion in the tumor microenvironment. Second, MIS18BP1 may modulate epigenetic programs governing T cell fate decisions independently of its canonical centromeric role. For example, chromatin accessibility at critical immune loci (such as FOXP3 and TBX21) is regulated by ATP-dependent remodeling complexes, and MIS18BP1 might interact with such machinery at non-centromeric sites in T cells. These interpretations remain speculative and require experimental validation through T cell-specific knockout models or chromatin immunoprecipitation assays.

The findings of this study raise hypothetical directions for clinical translation. It must be emphasized that all hypothetical suggestions below represent working hypotheses derived from statistical association and computational inference, not established facts supported by functional experiments or clinical validation. Any clinical application would require extensive preclinical verification. First, if future studies validate the exploratory MR findings, LMAN1 and MIS18BP1 may warrant investigation as potential genetic biomarkers for bladder cancer risk stratification in SLE patients, and enhanced urological malignancy screening might be considered for SLE patients carrying high-risk genotypes. Second, targeted intervention strategies directed against LMAN1-mediated vesicle transport pathways or MIS18BP1-related chromatin remodeling pathways might theoretically ameliorate immune dysregulation in SLE and immunotherapy resistance in bladder cancer. For instance, glycosylation inhibitors or vesicle transport modulators may restore T cell activation thresholds by reducing LMAN1 function; whereas histone deacetylase inhibitors or chromatin remodeling complex modulators may reestablish T cell differentiation balance by upregulating MIS18BP1 expression. From a hypothetical therapeutic perspective, glycosylation modulation or vesicle transport regulators could restore T cell activation thresholds by targeting LMAN1-mediated pathways; similarly, histone deacetylase inhibitors or chromatin remodeling modulators might reestablish T cell differentiation balance by modulating T cell epigenetic states. Recent studies have shown that reprogramming of N-glycosylation branching in CD8^+^ T cells can limit exhaustion and enhance cytotoxic function [46], and genome-wide CRISPR screens have confirmed that perturbation of chromatin remodeling complexes (e.g., BAF and INO80) improves the persistence of tumor-infiltrating lymphocytes and downregulates exhaustion-associated genes [47]. However, these therapeutic speculations are based primarily on computational and genetic inference and require extensive preclinical validation before any clinical translation. Furthermore, molecules identified through miRNA regulatory network analysis, such as hsa-miR-34a-5p (targeting LMAN1) and hsa-miR-524-5p (targeting MIS18BP1), provide hypothetical candidate targets for RNA interference or antisense oligonucleotide therapy. In summary, all proposed therapeutic and biomarker applications discussed above remain working hypotheses pending experimental validation. This study does not provide functional perturbation data, mechanistic evidence, or clinical outcomes. Until such evidence is obtained, these directions should be regarded as exploratory leads rather than actionable clinical strategies.

All findings in this study were derived from publicly available datasets through computational analyses, without independent experimental or clinical validation. This study has the following limitations. First, the Mendelian randomization analysis was based on GWAS summary data from European populations, and caution is warranted when extrapolating conclusions to other ethnic groups; future studies are needed to validate genetically predicted associations in East Asian, African, and other populations. Second, the single-cell datasets were derived from public databases with limited sample sizes (14,344 cells for SLE and 96,017 cells for bladder cancer), and independent validation cohorts were lacking; the expression characteristics of candidate genes require confirmation in larger-scale clinical samples. Third, MR analysis used gene expression as the exposure variable. If the true underlying pathway operates primarily at the protein level, but instrumental SNPs only influence mRNA stability or transcription rate without proportionally altering functional protein output, MR estimates may reflect transcriptional propensity rather than protein-mediated mechanisms. Because mRNA abundance typically explains only ~30–40% of protein variance in mammalian cells [23], this transcript–protein gap may introduce attenuation bias. In this study, LMAN1 lectin function depends on glycosylation, and MIS18BP1 activation relies on PLK1-mediated phosphorylation—features that transcriptomic MR cannot distinguish between active and inactive protein pools. Consequently, inferred protective or risk effects may be partially buffered by translational regulation. Future studies could bridge this gap through T cell–specific proteomics (e.g., CITE-seq or targeted mass spectrometry) and functional rescue experiments. Until such validation is completed, we conservatively interpret our MR results as a genetic prioritization framework rather than conclusive evidence of protein-driven mechanisms. Fourth, this study represents a secondary data analysis with an observational design, and the influence of residual confounding factors (such as environment-gene interactions) cannot be fully excluded. Fifth, pseudotime analysis is based on computationally inferred differentiation trajectories, and their biological authenticity requires further validation through lineage tracing experiments or in vitro differentiation systems. Sixth, an additional methodological limitation concerns the pre-filtering of candidate genes by cross-disease T cell differential expression prior to MR analysis. While this strategy reduced noise and computational burden, it may have excluded genes that are mechanistically relevant at the population level but do not meet differential expression thresholds in T cells. Moreover, the fact that none of the four nominated comorbidity genes (including LMAN1 and MIS18BP1) survived FDR correction for multiple testing indicates that this pre-filtering strategy, combined with an uncorrected *p*-value threshold, may have enriched for false-positive genetic associations. The nominated comorbidity genes should therefore be viewed as prioritized candidates within the T cell context rather than an exhaustive or definitive set of disease-relevant genes. Transparency regarding multiple-testing correction. We acknowledge that the initial MR screening using an uncorrected *p* < 0.05 threshold inflates the false-positive probability. After FDR correction (Benjamini–Hochberg, applied separately within each MR direction), no gene remained significant in both the SLE and bladder cancer directions; specifically, LMAN1 and MIS18BP1—our principal candidates—did not survive multiplicity correction. This underscores that the MR component of this study is hypothesis-generating rather than confirmatory. The sensitivity analyses (Cochran’s Q, MR-Egger intercept, leave-one-out, Steiger directionality) assess the internal robustness of individual MR estimates but do not account for the multiple-testing burden across the candidate gene set. Consequently, LMAN1 and MIS18BP1 should be viewed as biologically plausible exploratory candidates nominated by the intersection of cross-disease T cell DEGs and uncorrected MR estimates, and their putative mechanistic roles require independent validation through genome-wide MR without pre-filtering, replication in larger GWAS cohorts, and functional perturbation experiments in T cell models. The FDR-significant genes in single-disease directions (e.g., HLA-DRB1 for SLE, CFL1 for bladder cancer) represent additional leads for disease-specific investigation.

In summary, this study nominated LMAN1 and MIS18BP1 as exploratory prioritized candidate genes in SLE–bladder cancer comorbidity through integrated Mendelian randomization and single-cell transcriptome analysis, and suggests a hypothetical shared mechanism potentially associated with immune dysregulation through modulation of T cell differentiation trajectories. These findings are hypothesis-generating; the MR associations did not survive FDR correction for multiple testing, and the proposed roles require independent validation. This work may expand our understanding of the pathological basis of autoimmune–malignancy comorbidity and could provide a theoretical basis for developing cross-disease targeted intervention strategies, pending experimental confirmation.

## Data Availability

The scRNA-seq datasets are publicly available from the Gene Expression Omnibus (SLE: GSE266852; bladder cancer: GSE222315). GWAS summary statistics were obtained from the IEU OpenGWAS database (SLE: finn-b-L12_LUPUS; bladder cancer: ieu-b-4874). All data supporting the findings of this study are available from the corresponding author upon reasonable request.

## Supplementary Materials

The following supporting information can be downloaded at: https://www.mdpi.com/article/10.3390/life16081381/s1, Supplementary Materials S2: Workflow of the integrative bioinformatics analysis; Table S1: Heterogeneity test of SLE; Table S2: Horizontal pleiotropytest of SLE; Table S3: Steiger’s directionality test of SLE; Table S4: Heterogeneity test of Bladder cancer; Table S5: Horizontal pleiotropy test of Bladder cancer; Table S6: Steiger’s directionality test of Bladder cancer; Table S7: Pseudotime-binned expression of LMAN1 and MIS18BP1 in T cells from SLE and bladder cancer scRNA-seq datasets; Table S8: FDR-significant genes in single-disease MR directions for SLE and bladder cancer.

## Abbreviations

SLE	systemic lupus erythematosus
MR	Mendelian randomization
scRNA-seq	single-cell transcriptome sequencing
SIR	standardized incidence ratio
QC	Quality Control
OR	odds ratios
GO	Gene Ontology
DEGs	differentially expressed genes
GEO	Gene Expression Omnibus
GWAS	genome-wide association study
PCA	Principal component analysis
