# Supplementary material for: Integrated Mendelian Randomization and Single-Cell Transcriptomics Reveal T Cell Immune Mechanisms in Systemic Lupus Erythematosus–Bladder Cancer Comorbidity
Source: Life (Basel). 2026 Aug 21;16(8):1381. doi: 10.3390/life16081381 (PMC13514510; doi:10.3390/life16081381)
Supplement: Supplementary file 1 [file life-16-01381-s001.zip › Supplementary Materials S1.pdf]

TableS1: Heterogeneity test of SLE

exposure	method	Q	Q_df	Q_pval
ABCA2	Inverse variance weighted	14.9272873878656	20	0.780553010506885
ALKBH7	Inverse variance weighted	2.05201633452248	2	0.358434920835404
ARL6IP6	Inverse variance weighted	1.56604572903244	2	0.457022404321439
BSG	Inverse variance weighted	18.5030145251736	14	0.184821574657886
CD63	Inverse variance weighted	1.44329595511957	8	0.993609918463665
CDCA4	Inverse variance weighted	9.36161197678301	8	0.31271487329764
CHCHD2	Inverse variance weighted	46.0555758645069	62	0.935215447361676
CORO1A	Inverse variance weighted	2.00039651722938	4	0.735685947074984
CTPS1	Inverse variance weighted	12.0812209754632	21	0.937399147183646
DBI	Inverse variance weighted	6.0291855028536	10	0.812805270093304
DDX60	Inverse variance weighted	3.69938995017768	3	0.295807649656621
DNAJC1	Inverse variance weighted	3.77463092998918	4	0.437364515756354
EMP3	Inverse variance weighted	1.43437945359357	2	0.488122086679247
ENO1	Inverse variance weighted	9.98423315527508	11	0.531805950496918
EPSTI1	Inverse variance weighted	32.6286409915238	32	0.435892453896862
ETNK1	Inverse variance weighted	2.05888075248771	5	0.840939254226293
FBLN5	Inverse variance weighted	76.1839145690376	74	0.40808176201972
GBP3	Inverse variance weighted	76.6389587131499	76	0.457889631054745
GIT2	Inverse variance weighted	1.24017677486331	5	0.940965371684231
IL7R	Inverse variance weighted	11.7394239108444	21	0.946386715758452
INPP5D	Inverse variance weighted	15.889656955271	17	0.531670358749812
ITPK1	Inverse variance weighted	1.83369284814086	7	0.968469028567477
JAKMIP1	Inverse variance weighted	12.6110195370217	10	0.246240307548174
KIF20B	Inverse variance weighted	13.913858406135	12	0.306242945055973
LAP3	Inverse variance weighted	79.3055666632668	66	0.126007869361351
LINC00402	Inverse variance weighted	8.59922552078685	14	0.855836111986897
LMAN1	Inverse variance weighted	13.7872165280806	13	0.388998178771904
LMNB1	Inverse variance weighted	2.20579180996088	5	0.819998938986033
MCOLN2	Inverse variance weighted	21.5889291602827	33	0.936377705341389
MIS18BP1	Inverse variance weighted	9.61764452407969	11	0.565072297125201
MT1E	Inverse variance weighted	16.673616587859	21	0.730691085350147
MT1F	Inverse variance weighted	42.9939588313842	55	0.880144559594626
NDUFS8	Inverse variance weighted	41.6516014797457	33	0.143559031857758
NUP35	Inverse variance weighted	15.4373007316756	14	0.348929616883298
OSBPL3	Inverse variance weighted	22.1675141881979	32	0.902859389747097
PCED1B	Inverse variance weighted	40.0006589936211	40	0.470227996126636
PDIA6	Inverse variance weighted	6.25733748274871	12	0.902557935535482
POLR2E	Inverse variance weighted	10.4418744423426	16	0.84255704750761
PPP6R1	Inverse variance weighted	3.60452101671889	4	0.462164637814521
PRC1	Inverse variance weighted	5.55504172977273	7	0.592553365908801
PSMB3	Inverse variance weighted	17.8517805869342	19	0.53235858984066

RPL9	Inverse variance weighted	19.8498759090786	16	0.227058428655607
SESN1	Inverse variance weighted	33.4751936082007	27	0.181837675511863
SLC27A3	Inverse variance weighted	5.05641522826946	8	0.751529731185352
SLC40A1	Inverse variance weighted	51.5097885962308	38	0.0706166417891883
SNRPC	Inverse variance weighted	26.4053802462344	21	0.191411739363187
SP140	Inverse variance weighted	18.0004342772808	27	0.903506438747695
SQSTM1	Inverse variance weighted	15.9164763761095	20	0.72179222282963
SRGAP2	Inverse variance weighted	8.19229218352699	5	0.145951602692467
TAP1	Inverse variance weighted	0.750647479107248	2	0.687066812079226
TCIRG1	Inverse variance weighted	38.9847040426808	34	0.255508579214415
TFRC	Inverse variance weighted	14.5848931938042	34	0.998530165847421
TMED2	Inverse variance weighted	7.72921947467066	7	0.357075583435908
TTC38	Inverse variance weighted	6.71233008298159	18	0.992336215833629
WEE1	Inverse variance weighted	6.77528173537142	11	0.816978697164572

TableS2 Horizontal pleiotropytest of SLE

exposure	egger_intercept	se	pval
ABCA2	0.0637788206640903	0.0550918958586669	0.261340920683723
ALKBH7	-0.168231484	0.162618248371731	0.489200069509075
ARL6IP6	-0.199098638	0.159936601193696	0.430833660045669
BSG	0.0771811215314407	0.0579927632775582	0.206101862822265
CD63	0.0202692463353167	0.116285739763776	0.866559407770898
CDCA4	-0.007933437	0.0820293105088574	0.92566377932865
CHCHD2	0.00191229137292777	0.0241789656665916	0.9372206778381
CORO1A	0.0540074197355309	0.157016449529315	0.753573170415538
CTPS1	-0.031972149	0.0467622880794095	0.50199955458005
DBI	0.0258096332297487	0.0734086998591062	0.73323815712513
DDX60	-0.138072678	0.180301211432718	0.52383386141255
DNAJC1	-0.030520751	0.184668229132223	0.879238126653182
EMP3	0.141147353496782	0.171667947232353	0.561917396879035
ENO1	-0.03168738	0.0691073939200472	0.656379678656529
EPSTI1	-0.068648485	0.0406050066383943	0.100937839589779
ETNK1	-0.04890421	0.107907892801344	0.673898773386869
FBLN5	0.0334223162118982	0.0286885144915672	0.247809078867007
GBP3	0.0036264565304807	0.0274041663692191	0.895075841411195
GIT2	0.029261381170051	0.073964311393849	0.712582074084857
IL7R	0.0135853653273874	0.0533001034935646	0.801414958529066
INPP5D	-0.060314675	0.0510497263931685	0.254672572092383
ITPK1	0.0224870433672258	0.0662328672204993	0.745790720553961
JAKMIP1	0.00156462152180399	0.0871340135272474	0.986065337849129
KIF20B	0.0882284053411868	0.0683556345068134	0.223267312810224
LAP3	-0.001774002	0.0312705612198187	0.954933727117554
LINC00402	-0.029949074	0.060935176030006	0.631273218259714

LMAN1	0.0655024258552237	0.0602266975352729	0.298135138137433
LMNB1	-0.092696028	0.105865570218586	0.430677692566606
MCOLN2	0.00635996584459369	0.0374699719778668	0.866287061450833
MIS18BP1	0.00376038057532717	0.0705026184839643	0.958514082565029
MT1E	-0.008392082	0.0563265134544529	0.883053672825001
MT1F	-0.018674497	0.0265860633065272	0.485436246953809
NDUFS8	0.00506114182903281	0.0478144966970422	0.916362505649928
NUP35	0.0688123321408911	0.0820060578218086	0.41656989982948
OSBPL3	0.0326820223630206	0.0373209320094139	0.387927040397619
PCED1B	0.0465666785609839	0.0389977736222456	0.239656935051743
PDIA6	-0.00484341	0.0598019695719081	0.936904077777634
POLR2E	0.0442558946856459	0.0559496467456767	0.441275082300675
PPP6R1	-0.061385842	0.083955042091747	0.517605580321691
PRC1	-0.060817431	0.0760537052766315	0.454390633684301
PSMB3	-0.027314172	0.0615429810307719	0.662459898728435
RPL9	0.0318773468928805	0.0590288273600642	0.597103291967877
SESN1	-0.030590894	0.0360882600438426	0.404358479238105
SLC27A3	0.0737790754115183	0.0595505945766984	0.255299545037887
SLC40A1	-0.063224239	0.0687483364916569	0.363714039024502
SNRPC	0.0531284646390065	0.0561750156015875	0.355554719684214
SP140	0.0577062963212047	0.0556132915714849	0.308994449475578
SQSTM1	-0.035923701	0.0587722660169684	0.54828756330459
SRGAP2	0.23250729946428	0.0912164501981637	0.0633751275121891
TAP1	0.072081772651957	0.204272015628843	0.78403905761785
TCIRG1	0.00153958516090932	0.0520581317994751	0.97658466529935
TFRC	-0.021620523	0.0361333677300206	0.55368919765858
TMED2	0.0082850908153102	0.0969227965714104	0.934659793009557
TTC38	0.0192176691534516	0.0446509883007873	0.672313424955982
WEE1	-0.00470876	0.0654731088008458	0.944084452252202

TableS3 Steiger's directionality test of SLE

exposure	outcome	correct_causal_direction	steiger_pval
ABCA2	SLE	TRUE	3.95656736934289e-291
ALKBH7	SLE	TRUE	4.71161700215521e-52
ARL6IP6	SLE	TRUE	1.24419258148631e-10
BSG	SLE	TRUE	9.42604935624952e-256
CD63	SLE	TRUE	4.28222856098471e-54
CDCA4	SLE	TRUE	1.14707551920972e-77
CHCHD2	SLE	TRUE	NA
CORO1A	SLE	TRUE	7.20987529073866e-29
CTPS1	SLE	TRUE	0
DBI	SLE	TRUE	8.19578201331299e-200

DDX60	SLE	TRUE	7.78266692231733e-10
DNAJC1	SLE	TRUE	1.96560048903554e-11
EMP3	SLE	TRUE	1.00891642406675e-05
ENO1	SLE	TRUE	5.01209359732194e-133
EPSTI1	SLE	TRUE	0
ETNK1	SLE	TRUE	1.04453431815009e-42
FBLN5	SLE	TRUE	0
GBP3	SLE	TRUE	0
GIT2	SLE	TRUE	7.91856561253313e-133
IL7R	SLE	TRUE	0
INPP5D	SLE	TRUE	4.50208584439119e-171
ITPK1	SLE	TRUE	5.03737792791694e-137
JAKMIP1	SLE	TRUE	8.51641069372178e-209
KIF20B	SLE	TRUE	3.77312893800019e-85
LAP3	SLE	TRUE	0
LINC00402	SLE	TRUE	0
LMAN1	SLE	TRUE	0
LMNB1	SLE	TRUE	2.47530151023878e-156
MCOLN2	SLE	TRUE	0
MIS18BP1	SLE	TRUE	1.20290463432743e-86
MT1E	SLE	TRUE	0
MT1F	SLE	TRUE	0
NDUFS8	SLE	TRUE	0
NUP35	SLE	TRUE	3.6698452210099e-132
OSBPL3	SLE	TRUE	0
PCED1B	SLE	TRUE	0
PDIA6	SLE	TRUE	2.87423845421539e-237
POLR2E	SLE	TRUE	1.64985615716716e-206
PPP6R1	SLE	TRUE	6.29167931547612e-29
PRC1	SLE	TRUE	1.72698918958702e-58
PSMB3	SLE	TRUE	6.63434545686987e-308
RPL9	SLE	TRUE	5.66563437813661e-169
SESN1	SLE	TRUE	0
SLC27A3	SLE	TRUE	1.25072354221899e-181
SLC40A1	SLE	TRUE	0
SNRPC	SLE	TRUE	9.3051340280994e-205
SP140	SLE	TRUE	8.27441383200184e-283
SQSTM1	SLE	TRUE	8.30181849321598e-185
SRGAP2	SLE	TRUE	1.1115658726662e-53
TAP1	SLE	TRUE	2.50687075781367e-10
TCIRG1	SLE	TRUE	0
TFRC	SLE	TRUE	0
TMED2	SLE	TRUE	7.57074659460915e-29
TTC38	SLE	TRUE	0

WEE1	SLE	TRUE	6.0976814638262e-123

TableS4 Heterogeneity test of Bladder cancer

exposure	method	Q	Q_df	Q_pval
ABHD2	Inverse variance weighted	6.35892154739075	6	0.384206819801958
ACTN4	Inverse variance weighted	2.66622005085793	10	0.9882258272807
ANKRD10	Inverse variance weighted	16.6260593111011	28	0.95561426358547
ANXA6	Inverse variance weighted	32.5756333693319	37	0.676519947255448
CCR4	Inverse variance weighted	10.4820382701487	12	0.573749497310903
CD151	Inverse variance weighted	74.6786611375667	66	0.217090330463232
CD3D	Inverse variance weighted	0.290401118434461	3	0.961823545017868
CEP68	Inverse variance weighted	9.59768211863184	15	0.844255139449021
CFL1	Inverse variance weighted	9.98297513432961	11	0.531919202820281
CKS2	Inverse variance weighted	6.91930195681668	15	0.959850448567408
CLIC3	Inverse variance weighted	8.36176984386736	12	0.756258954153496
CORO1A	Inverse variance weighted	1.99906684730349	3	0.572600406400951
CST7	Inverse variance weighted	24.2369158535196	34	0.892150564283526
DEF6	Inverse variance weighted	44.2061665083847	42	0.378610742054607
ERAP1	Inverse variance weighted	10.8294174516395	15	0.764590274840404
FAM111A	Inverse variance weighted	6.17888911852644	4	0.186181103730794
GBP3	Inverse variance weighted	69.4031429752952	75	0.660584082137019
GZMA	Inverse variance weighted	5.72008102852405	6	0.455265701255878
HLA-A	Inverse variance weighted	24.1331862428091	25	0.51168884780113
HSP90AA1	Inverse variance weighted	8.70627069242523	12	0.727792925094618
IL32	Inverse variance weighted	46.37376074	35	0.0946173144393519
LDHA	Inverse variance weighted	1.09265215033977	4	0.895436265603955
LGALS1	Inverse variance weighted	8.94665590849436	14	0.834452539644149
LMAN1	Inverse variance weighted	12.4111610144711	12	0.413248886310633
MIF	Inverse variance weighted	5.47955675411723	11	0.905740674142978
MIS18BP1	Inverse variance weighted	9.56335640784509	12	0.654206867981477
MRPL22	Inverse variance weighted	1.94415668735826	5	0.856816829970507
NEDD9	Inverse variance weighted	2.18371256492036	7	0.948976295099777
NOSIP	Inverse variance weighted	6.62660061945194	7	0.468764490794598
PCMT1	Inverse variance weighted	11.7363468645071	20	0.92485179604261
PHF19	Inverse variance weighted	27.300591708561	22	0.200031479256856
PRPF19	Inverse variance weighted	2.88321002439446	6	0.823356042528849
PSMA4	Inverse variance weighted	4.30924018322799	7	0.743549270599006
RASSF4	Inverse variance weighted	35.158524246002	30	0.236918037835098
RFWD3	Inverse variance weighted	33.8693614575019	37	0.616605477987532
RNF167	Inverse variance weighted	45.1131859122703	39	0.231583914899432
RPS14	Inverse variance weighted	6.04108336091423	13	0.944644951001547
RPS18	Inverse variance weighted	11.6901600335183	9	0.231345464584095

RPS5	Inverse variance weighted	11.658412700898	13	0.555833085254155
RPS8	Inverse variance weighted	18.3393787612753	14	0.191758019199834
SAMD3	Inverse variance weighted	39.1594895472713	39	0.462719580369374
SERINC5	Inverse variance weighted	22.1211582705618	18	0.226650499414736
SLC1A5	Inverse variance weighted	12.5197068443037	13	0.485556297495349
SLC25A24	Inverse variance weighted	47.1224141584751	38	0.147343856572407
SLC35B2	Inverse variance weighted	1.62965600497122	6	0.950421714283234
SLC40A1	Inverse variance weighted	19.026562636001	37	0.993692392699457
SMAP2	Inverse variance weighted	2.1678627862043	4	0.704917016260921
SYNGR2	Inverse variance weighted	5.27149223436041	7	0.626869494887422
TMEM109	Inverse variance weighted	11.9925001175711	14	0.606905109513852
TMEM14A	Inverse variance weighted	5.87809941262131	9	0.752053944570858
TNF	Inverse variance weighted	15.3568290329767	14	0.354205517967259
TPM3	Inverse variance weighted	14.4759461528268	9	0.106373616541471
UBA52	Inverse variance weighted	22.1604097961303	17	0.178628516136816
UBE2L6	Inverse variance weighted	1.38025898679234	2	0.501511122482601
ZMPSTE24	Inverse variance weighted	5.34999929885678	8	0.719596059884324

TableS5 Horizontal pleiotropy test of Bladder cancer

exposure	egger_intercept	se	pval
ABHD2	0.000289394762229184	0.000238209171727309	0.278648938696763
ACTN4	5.41688095155778e-05	0.000145628552783316	0.718525504291935
ANKRD10	-1.00E-07	9.1635000829817e-05	0.999134292510264
ANXA6	-3.03E-05	6.38589008842841e-05	0.637559861677803
CCR4	-3.96E-05	0.00010530404480364	0.713819425746113
CD151	2.14161464407697e-05	4.28844394212943e-05	0.619189430774262
CD3D	-9.64E-05	0.000198396620918567	0.675024136355752
CEP68	-9.74E-05	0.00012033563989914	0.431745086227723
CFL1	0.000155753356666587	0.000101394382484478	0.155523564970714
CKS2	-8.10E-05	7.12583763471131e-05	0.274807396871591
CLIC3	-7.98E-05	0.0001576284987365	0.622705753137908
CORO1A	-3.92E-05	0.00027709499835337	0.900351828178869
CST7	-1.65E-06	5.29456409739609e-05	0.975389457545816
DEF6	-4.79E-05	6.53283586403335e-05	0.467983509233602
ERAP1	-3.41E-06	0.000142576494254299	0.981259732437019
FAM111A	3.23223609633647e-05	0.000251398765071329	0.905832930610388
GBP3	-5.25E-05	4.13518899659593e-05	0.208295219660804
GZMA	-2.81E-05	0.000163691042799271	0.870356296684978
HLA-A	-7.57E-05	0.000131169848474711	0.569033328220864
HSP90AA1	9.26756222241596e-05	0.000110690917668788	0.420272150401507
IL32	-8.13E-05	6.90444803184356e-05	0.246916793824407
LDHA	-9.00E-05	0.000197727603066096	0.679882014096157
LGALS1	-0.000161645	8.17543824553402e-05	0.0696209500268816
LMAN1	0.000154965498898603	0.000106426324283647	0.173311603307507

MIF	4.76776782285527e-05	0.000116584111709152	0.691193405801797
MIS18BP1	-0.000122247	0.000106517072966593	0.275450741072952
MRPL22	-7.25E-05	0.000171504313626749	0.694059964290003
NEDD9	-0.000114417	0.000141278914409063	0.44893586695702
NOSIP	4.49690056738755e-05	0.000188099619807517	0.819007949442067
PCMT1	9.02472383006519e-06	8.75154682682153e-05	0.918947222616468
PHF19	-0.000112686	0.000183139764157082	0.544968508999636
PRPF19	0.000190836367044684	0.000267717728476618	0.507828547814362
PSMA4	-0.000128185	0.000153544567451797	0.435780905007914
RASSF4	4.03843724818932e-05	6.59233940251873e-05	0.54491807496075
RFWD3	5.05549102026854e-05	7.29813954358469e-05	0.492936517448631
RNF167	-7.02E-05	6.09317610305312e-05	0.256534079084572
RPS14	5.88422156205591e-05	0.000142736988024505	0.687430917
RPS18	-3.82E-05	0.000112127479677991	0.74189816850387
RPS5	-7.98E-05	0.000143441759940077	0.588039190717547
RPS8	0.000154072062603236	8.39586570219994e-05	0.0894687953203125
SAMD3	-6.15E-05	7.21806210591294e-05	0.399434427621092
SERINC5	-0.000143902	8.49916400175576e-05	0.108673997358438
SLC1A5	0.000133805590502607	0.000169231656000014	0.44448507593301
SLC25A24	1.10216856748272e-05	8.40092122427566e-05	0.896330920921469
SLC35B2	5.34877887137703e-05	0.000126122894380355	0.68912562292409
SLC40A1	3.55080437018693e-05	0.000107001664959825	0.741930081985521
SMAP2	-0.000241895	0.000395317279478929	0.583856436801199
SYNGR2	-1.21E-05	0.000255370984962967	0.963682495346789
TMEM109	4.60667910123667e-05	0.000104470053901455	0.666488560780294
TMEM14A	0.000116291330695962	0.00011131964640526	0.326715340570089
TNF	7.01533477103719e-05	0.000111915517858548	0.541615195425035
TPM3	4.35867721715685e-05	0.000163679781148315	0.796748359660351
UBA52	0.000109289557423203	0.000150728683426108	0.478877194574519
UBE2L6	-0.000141336	0.000256246305003727	0.679117030267142
ZMPSTE24	7.22505278516168e-05	0.000184421409270298	0.706882437774084

TableS6 Steiger's directionality test of Bladder cancer

exposure	outcome	correct_causal_direction	steiger_pval
ABHD2	outcome	TRUE	1.34056100614336e-146
ACTN4	outcome	TRUE	2.98163990055328e-207
ANKRD10	outcome	TRUE	0
ANXA6	outcome	TRUE	0
CCR4	outcome	TRUE	0
CD151	outcome	TRUE	0
CD3D	outcome	TRUE	6.45455163357236e-70
CEP68	outcome	TRUE	0
CFL1	outcome	TRUE	2.0682245092349e-244
CKS2	outcome	TRUE	0

CLIC3	outcome	TRUE	1.15299315877268e-176
CORO1A	outcome	TRUE	2.5222046537134e-80
CST7	outcome	TRUE	0
DEF6	outcome	TRUE	0
ERAP1	outcome	TRUE	0
FAM111A	outcome	TRUE	4.60951172247397e-126
GBP3	outcome	TRUE	0
GZMA	outcome	TRUE	2.06137733633667e-106
HLA-A	outcome	TRUE	0
HSP90AA1	outcome	TRUE	0
IL32	outcome	TRUE	0
LDHA	outcome	TRUE	1.39267603361672e-132
LGALS1	outcome	TRUE	0
LMAN1	outcome	TRUE	0
MIF	outcome	TRUE	3.49624918216671e-213
MIS18BP1	outcome	TRUE	1.1038565051378e-245
MRPL22	outcome	TRUE	1.69564435817872e-143
NEDD9	outcome	TRUE	1.00560743017105e-209
NOSIP	outcome	TRUE	1.48175554866986e-198
PCMT1	outcome	TRUE	0
PHF19	outcome	TRUE	0
PRPF19	outcome	TRUE	5.47593050858942e-170
PSMA4	outcome	TRUE	9.32186946667823e-159
RASSF4	outcome	TRUE	0
RFWD3	outcome	TRUE	0
RNF167	outcome	TRUE	0
RPS14	outcome	TRUE	2.51579600808734e-259
RPS18	outcome	TRUE	1.35113231395151e-274
RPS5	outcome	TRUE	0
RPS8	outcome	TRUE	0
SAMD3	outcome	TRUE	0
SERINC5	outcome	TRUE	0
SLC1A5	outcome	TRUE	1.46803701611393e-318
SLC25A24	outcome	TRUE	0
SLC35B2	outcome	TRUE	1.44353138423594e-138
SLC40A1	outcome	TRUE	0
SMAP2	outcome	TRUE	4.77316211771019e-78
SYNGR2	outcome	TRUE	5.64689492822881e-147
TMEM109	outcome	TRUE	0
TMEM14A	outcome	TRUE	3.06777452698793e-193
TNF	outcome	TRUE	5.23647332320345e-318
TPM3	outcome	TRUE	8.09715349004811e-163
UBA52	outcome	TRUE	0
UBE2L6	outcome	TRUE	2.83550597946937e-87

ZMPSTE24	outcome	TRUE	2.75639067142275e-139

Table S7. Pseudotime-binned expression of LMAN1 and MIS18BP1 in T cells from SLE and bladder cancer scRNA-seq datasets.

Dataset	Decile	n cells	LMAN1 (mean)	MIS18BP1 (mean)
SLE (GSE266852)	1 (early)	857	0.276	0.177
SLE (GSE266852)	2	857	0.241	0.161
SLE (GSE266852)	3	857	0.320	0.177
SLE (GSE266852)	4	857	0.321	0.191
SLE (GSE266852)	5	856	0.340	0.199
SLE (GSE266852)	6	856	0.372	0.263
SLE (GSE266852)	7	856	0.393	0.328
SLE (GSE266852)	8	856	0.386	0.429
SLE (GSE266852)	9	856	0.462	0.453
SLE (GSE266852)	10 (late)	856	0.497	0.490
Bladder cancer (GSE222315)	1 (early)	1641	0.185	0.233
Bladder cancer (GSE222315)	2	1640	0.301	0.212
Bladder cancer (GSE222315)	3	1640	0.256	0.198
Bladder cancer (GSE222315)	4	1640	0.299	0.265
Bladder cancer (GSE222315)	5	1640	0.326	0.222
Bladder cancer (GSE222315)	6	1640	0.320	0.266
Bladder cancer (GSE222315)	7	1640	0.366	0.285
Bladder cancer (GSE222315)	8	1640	0.452	0.320
Bladder cancer (GSE222315)	9	1640	0.559	0.409
Bladder cancer (GSE222315)	10 (late)	1640	0.647	0.861

Supplementary Table S8. FDR-significant genes in SLE and bladder cancer

Direction	Gene	method	nsnp	pval	or	fdr_pval
Bladder cancer	CFL1	Inverse variance weighted	12	5.50205735474705e-05	1.00132894218224	0.0298761714362765
SLE	HLA-DRB1	Inverse variance weighted	38	2.01391358658961e-05	1.30993801173006	0.00551812322725553
SLE	MT1F	Inverse variance weighted	56	0.000623362766213347	1.34746436463485	0.0488003994121306
SLE	TCIRG1	Inverse variance weighted	35	0.000391357973698633	0.66474017284593	0.0357440282644751
SLE	SLC27A3	Inverse variance weighted	9	7.63999550331423e-05	2.7325094016443	0.0104667938395405
SLE	TXN	Inverse variance weighted	16	0.000336212867083751	0.513622818071834	0.0357440282644751
SLE	SP140	Inverse variance weighted	28	1.49836019292382e-07	2.42012620350991	8.21101385722253e-05
SLE	ZMPSTE24	Inverse variance weighted	9	6.45508142324673e-05	4.86043177333987	0.0104667938395405
SLE	SMAP2	Inverse variance weighted	5	0.00071367090090271	5.43603473952487	0.0488864567118356
